# Clustering of childhood cancer in Colombia: a nationwide study

**DOI:** 10.12688/f1000research.27766.1

**Published:** 2021-02-09

**Authors:** Edgar F. Manrique-Hernández, Marcela Pilar Rojas Díaz, Laura Andrea Rodriguez-Villamizar

**Affiliations:** 1 Public Health Department, School of Medicine, Universidad Industrial de Santander. Bucaramanga, Bucaramanga, Colombia; 2 Instituto Nacional de Salud, Bogotá D.C., Colombia

**Keywords:** Cluster Analysis, Neoplasms, Childhood, Epidemiology, Colombia

## Abstract

**Background:** Childhood cancer is considered one the most important causes of death in children and adolescents, despite having a low incidence in this population. Spatial analysis has been previously used for the study of childhood cancer to study the geographical distribution of leukemias. This study aimed to identify the presence of space-time clusters of childhood of cancer excluding leukemia in Colombia between 2014 and 2017.

**Methods:** All incident cancer cases (excluding leukemia) in children under the age of 15 years that had been confirmed by the National Surveillance System of Childhood Cancer between 2014 and 2017 were included. Kulldorf’s circular scan test was used to identify clusters using the municipality of residence as the spatial unit of analysis and the year of diagnosis as the temporal unit of analysis. A sensitivity analysis was conducted with different upper limit parameters for the at-risk population.

**Results**: A total of 2006 cases of non-leukemia childhood cancer were analyzed, distributed in 432 municipalities with a mean annual incidence rate of 44 cases per million children under the age of 15. Central nervous system (CNS) tumors were the most frequent type. Four spatial clusters and two space-time clusters were identified in the central and southwest regions of the country. In the analysis for CNS tumors, a spatial cluster was identified in the central region of the country.

**Conclusions: **The distribution of non-leukemia childhood cancer seems to have a clustered distribution in some Colombian regions that may suggest infectious or environmental factors associated with its incidence.

## Introduction

Childhood cancer (CC) is considered one the most important causes of death in children and adolescents, despite having a low incidence in this population. The mean annual incidence of CC was estimated at 140.6 cases per million children between the age 0–14 years in the period of 2001 to 2010 ^1^. The world health organization (WHO) estimates that nearly 300,000 new cases of CC are diagnosed every year in children between 0 and 19 years of age ^2^. In the Americas it has been estimated that every year there are approximately 27,000 new cases of cancer in children under the age of 14 years, with an estimated mortality rate of 10,000 deaths/year ^3^. The majority of the incident cases in the Americas belong to the Latin American and Caribbean region making up nearly 65% of the diagnosed cases ^3^.

CC is a set of diseases that does not have a clear etiology yet. There are several conditions that have been identified as risk factors which include genetic factors, some infectious diseases, exposure to pesticides, benzene and radiation, alcohol consumption during pregnancy, smoking, and the socioeconomic condition of the family ^4, 5^. Some of these factors are more specific than others, as was found with Burkitt´s and Hodgkin´s lymphoma, where the Epstein-Barr virus plays a relevant role. However, there are still controversies surrounding the etiology of these diseases ^5^.

Spatial analysis allows the identification of geographical patterns of health and disease related events that point out variations between populations contributing to the generation of hypotheses about possible etiologies ^6^. Spatial analysis has been previously used for the study of CC, mainly for studying the geographical distribution of leukemias ^4, 7^, since this type of analysis allows for the identification of space and time variations in a geographical area that generate clusters that indicate an increase in the tendency of the cases ^4^. Clusters of acute childhood leukemia have been identified in Colombia ^8^, but analyses for CC other than leukemia are scarce ^5^. The objective of this study was to perform an exploratory study with space-time aggrupation to identify clusters of incident cases of CC other than leukemia in Colombian municipalities between 2014 and 2017. 

## Methods

### Population

Colombia is a country located in the north of South America, which limits with Venezuela and Brazil to the east, Panama to the Northwest, Peru and Ecuador to the south; it limits to the Caribbean sea with Panama, Costa Rica, Nicaragua, Honduras, Jamaica, Haiti, Dominican Republic, Venezuela and to the Pacific ocean with Panama, Costa Rica and Ecuador. The Colombian population for 2018 was approximately 48 million people ^9^. Women make up 51.2% of the population, and children under the age of 15 years make up 22.6% compared to adults over the age of 65 years which represent 9.1%. Most of the Colombian population live in urban areas (77.1%) ^9^.

### Cancer and population data sources

All incident cases of non-leukemia CC diagnosed in children under 15 years of age between 2014 and 2017 were included. The data source was the National Surveillance System for Public Health (SIVIGILA, for its name in Spanish) ^10^, which registers the newly confirmed and probable cases of CC in a systematic and mandatory manner. Surveillance for CC started in Colombia in 2008 with the registry of childhood leukemia cases and starting in 2013 the system registers all types of CC ^11^. SIVIGILA verifies the confirmation of reported cases according to the results of diagnostic tests such as myelograms, immunotyping, histopathology or cytogenetic tests; adjusting the real number of confirmed cases and the diagnosis date. De-identified non-leukemia CC data were provided by the National Health Institute (INS for its name in Spanish), allowing access to the following variables: municipality of residence, date of birth, diagnosis date and type of CC according to the International Classification of Childhood Cancer, Third Edition (ICCC-3). Cases were assigned a consecutive number which cannot be used to identify cases. SIVIGILA is the most complete registry of CC in Colombia, taking into account that it has a nationwide coverage and the reports are updated weekly ^8^. 

Data from CNS and miscellaneous intracranial and intraspinal neoplasms (Group III) cases according to the ICCC-3 ^12^ was extracted for a sub-analysis. This group is the second with the highest incidence after leukemias ^5, 13^.

Data for the at-risk population in the 1122 municipalities of Colombia was provided by the National Department of Statistics (DANE for its name in Spanish) ^10^ which performed its last national census in 2018. For the calculation of the population between the years 2014 and 2017 the dynamics of DANE projections of population was used, and an interpolation of the population was conducted for each one of the municipalities for previous years ^14^. The calculation of the coordinates (longitude and latitude) of the centroid of each municipality was done in QGIS version 3.16.3 using free cartographic information from the DANE Geoportal ^15^.

### Statistical analysis

We performed a descriptive analysis calculating frequencies and central tendency measurements. The incidence of CC was calculated for each municipality and a direct standardization by age and sex of the incidence rates was conducted using as reference the structure of children population for Colombia in 2017. Standardized rates and their respective confidence interval were obtained through STATA® version 14. The global Moran index was calculated to estimate the spatial autocorrelation. Choroplethic maps were built in order to visualize the standardized rates using the WGS84 projection for Colombia and the cartographic archives available for each municipality in the DANE cartography site using QGIS version 3.16.3 ^15^.

Kuldorff’s circular scan test was used to identify spatial and spatio-temporal clusters ^16^, using the SaTScan® software version 9.6. This is a spatial hypothesis test that runs consecutive scans in the study area with different circumference radii that increase in size; the null hypothesis of the test is that the risk of the event (in this case risk of non-leukemia CC) within the circle is the same as outside the scanned area. Space and space-time exploratory analysis were run using a Poisson distribution and scanning for high rates; the space analysis unit was the municipality of residence and the time analysis unit was the year of diagnosis. We used an upper limit of the population at risk of 25% and for a sensitivity analysis we assess the results using upper limits of 50% and 10%.

### Ethical approval

This research received ethical approval from the ethics committee of scientific research at the Universidad Industrial de Santander (CEINCI UIS), on October 27, 2017 (approval number 24-2017).

## Results

### Study population

**Figure 1.**
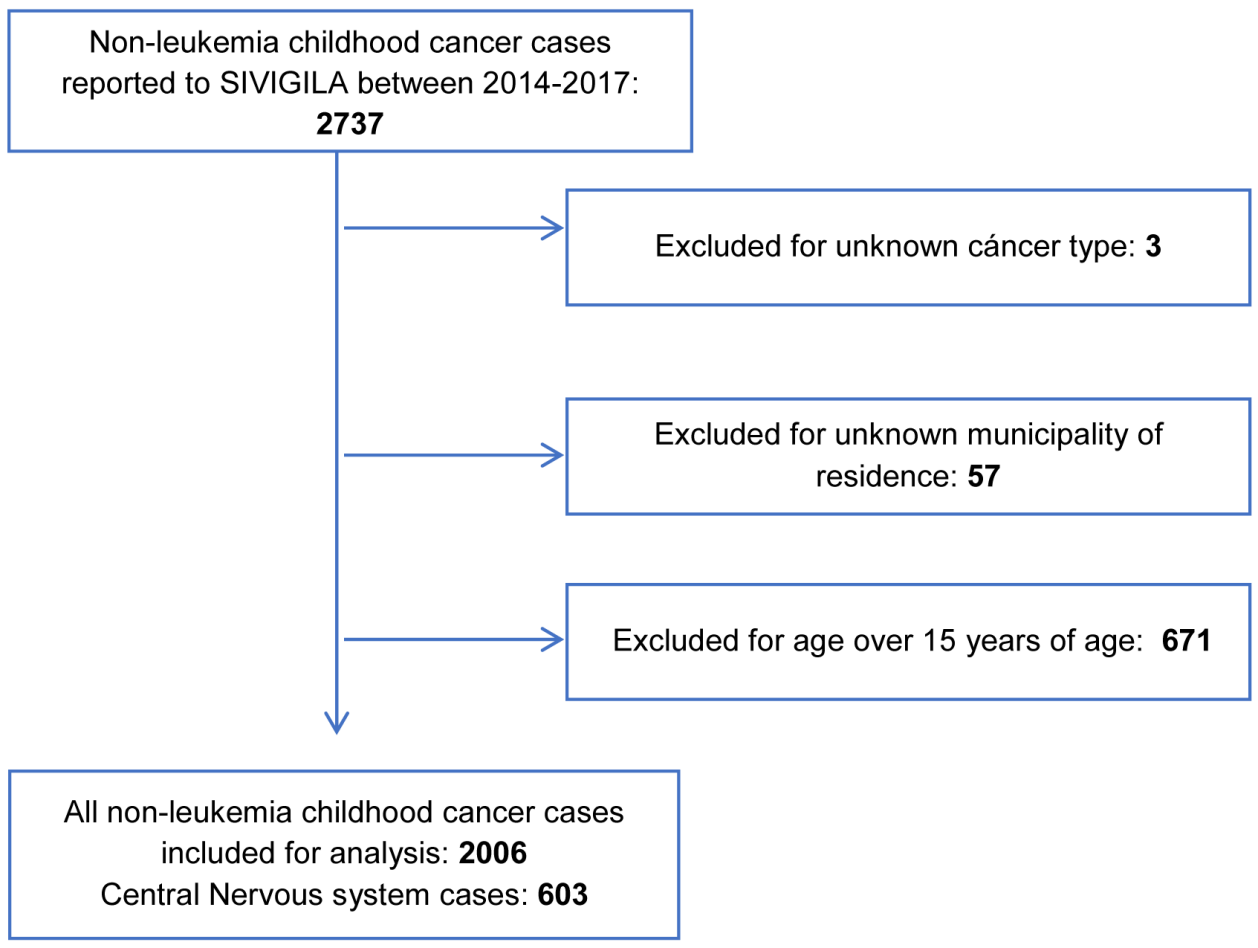
Study population selection flow chart.

SIVIGILA reported 2737 cases of non-leukemia CC between January 1st 2014 and December 31st 2017. A total of 731 cases were excluded for different reasons ( Figure 1). Therefore, a total of 2006 cases were included for the analysis, which were reported in 432 of the 1122 municipalities of Colombia (38.5%). Subsequently, for the analysis of CNS tumors those who were included in the ICCC-3 classification group III were selected, obtaining 603 cases reported in 201 municipalities (17.9%).

**Figure 2.**
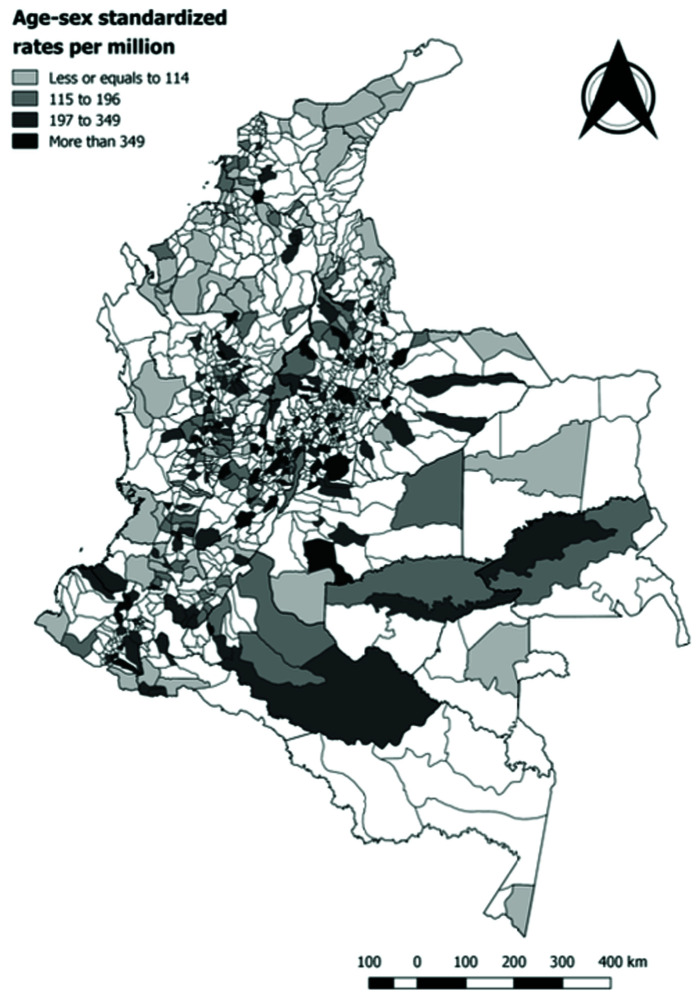
Standardized rates of non-leukemia childhood cancer by municipality, Colombia 2014–2017.

A slight majority of reported cases corresponded to males (54.74%) and 70.49% were reported in children under 9 years of age (0–4 years 33.5%, 5–9 years 36.99%, 10–14 years 29.51%). The mean annual incidence rate of non-leukemia CC was of 44 cases per million children under 15 years of age between 2014 and 2017 in Colombia. The highest incidence rates were reported in Meta (Villavicencio), Bogota D.C., Santander (Bucaramanga, Floridablanca), Bolivar (Cartagena), Valle del Cauca (Cali), Antioquia (Medellin), Cundinamarca (Soacha), Nariño (Pasto). The standardized rates by age and sex varied between 0 and 198 cases per million inhabitants under 15 years of age ( Figure 2). The Moran index was of 0.0023 (p=0.211) which indicates a low spatial autocorrelation of the incidence rates across Colombian municipalities.

For CNS tumors, again the slight majority of cases were reported in the male population (55.39%) and the 71.97% of the cases were reported in children under the age of 9 (0–4 years, 30.35%; 5–9 years, 41.63%; 10–14 years, 28.03%). The departments with the highest number of cases were Bogota D.C, Valle del Cauca (Cali and Palmira), Antioquia (Medellin), Bolivar (Cartagena), Meta (Villavicencio), Santander (Bucaramanga), Cundinamarca (Soacha) and Nariño (Pasto).

### Clustering results

**Figure 3.**
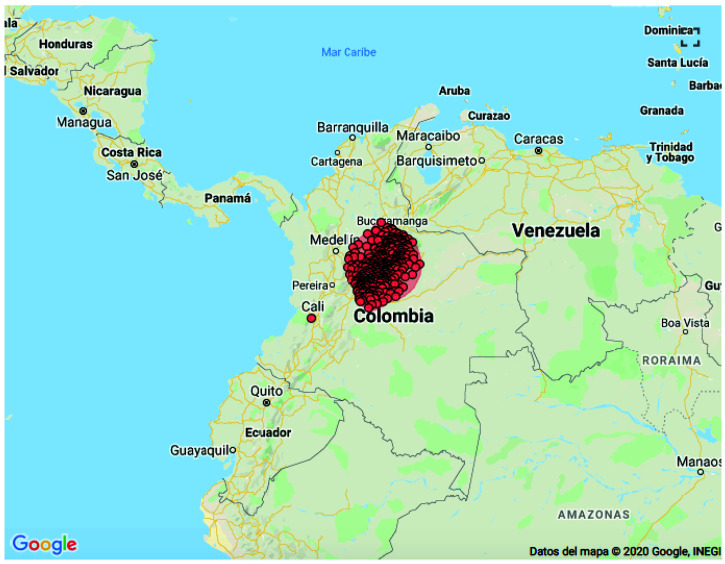
Spatial clusters of non-leukemia childhood cancer in Colombia, 2014–2017.

We identified four clusters in the spatial analysis for non-leukemia CC ( Figure 3). The first cluster in the central region of the country included 327 municipalities distributed in the following departments: Cundinamarca (95), Meta (6), Boyaca (122), Santander (76), Antioquia (7), Caldas (4), Casanare (13), Tolima (3) and Bogota D.C

This cluster has a radius of 172.11 Km and 798 cases, with an expected number of cases of 497.88 with a relative risk (RR)=2 and p value <0.0001. The second cluster was identified in the departments of Cundinamarca (57), Meta (7) and Bogota D.C. with a radius of 72.04 km, and superposition with the first cluster; in this cluster 623 cases were identified for a total of 358.29 expected cases with RR = 2.07 and p value <0.0001. The third cluster was identified in the southwest region of Colombia, corresponding to the city of Cali (Valle del Cauca) and without superposition with any other cluster. In this third cluster 152 cases were identified where 87.95 cases were expected, with a RR = 1.79 and a p value <0.0001. The fourth cluster was identified in 23 municipalities of the Santander (Northeast region) department, with a 58.50 km radius, super positioning with some municipalities from the first cluster; in this cluster there were 106 cases with 55.15 as the expected number of cases, obtaining a RR = 1.97 and a p value <0.0001.

Two clusters were identified in the space-time analysis for non-leukemia CC. The first cluster was located in the central region of the country corresponding to the following departments: Boyaca (122), Santander (76), Cundinamarca (95), Casanare (13), Meta (6), Caldas (4), Antioquia (7), Tolima (3), Bogota D.C. This cluster was identified between 2015 and 2016 with a radius of 172.11 Km, 491 cases reported with 249 expected obtaining a RR = 2.29 and p value <0.0001. The second cluster was identified in the city of Cali between 2016 and 2017, with 97 cases reported and 43.7 expected obtaining a RR = 2.28 and p value <0.0001.

**Figure 4.**
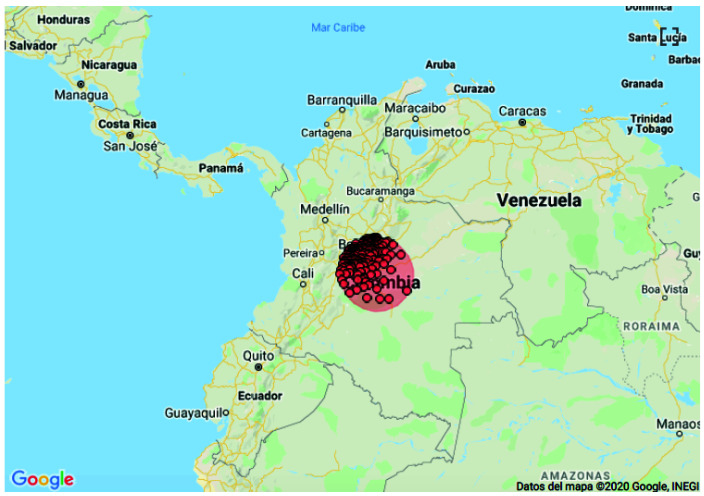
Spatial clusters of central nervous system cancers in Colombia, 2014–2017.

The spatial analysis for CNS tumors identified one cluster in the following departments: Meta (27), Cundinamarca (86), Casanare (8), Huila (1), Tolima (8) and Bogota D.C. ( Figure 4). This cluster has a radius of 177.43 km, with 237 cases reported and 122 expected for a RR = 2.55 and a p value <0.0001. The space-time analysis for CNS identified the same cluster in the central region corresponding to the following municipalities: Meta (16), Cundinamarca (95), Boyaca (30), Casanare (3), Tolima (1) and Bogota D.C. This cluster was identified between 2015 and 2016 with a radius of 112.53 km, with 143 cases reported and 58.9 expected obtaining a RR = 2.87 and p values <0.0001

### Sensitivity analysis

**Figure 5.**
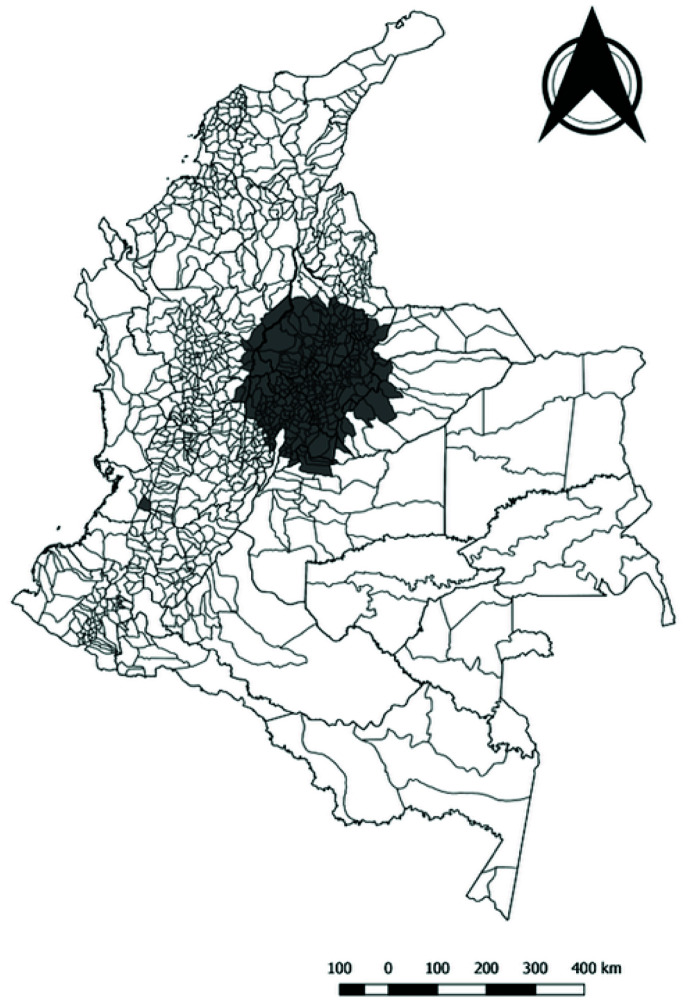
Municipalities consistently identified within spatial clusters of non-leukemia childhood cancer in Colombia, 2014–2017.

In the sensitivity analysis for non-leukemia CC circular scan tests were run using values of the at-risk population of 10% and 50%. There were 304 identified municipalities in the central region of the country that showed consistency in the three analysis (using 10%, 25% and 50% upper limit of at-risk population) ( Figure 5).

## Discussion

This study identified the presence of non-leukemia CC clusters between 2014 and 2017 in Colombia, using information with nationwide coverage available in SIVIGILA. To our knowledge, this is the first nation-wide study in South America using spatial analysis to describe the distribution and clustering of non-leukemia CC.

Spatial and spatio-temporal analysis have been previously used in this area, mainly in the study of the geographic pattern of leukemias ^7, 17^. A recent systematic review of space-time analysis identified 70 studies published up to 2016 of which 47 reported results for leukemias, 26 for lymphomas, 13 for CNS tumors and 12 for other types of tumors ^18^. All 32 analyses used for the meta-analysis were from Europe and United States; this analysis showed evidence of leukemia clustering in children between 0 and 5 years of age. However, the evidence was not conclusive for lymphomas and CNS tumors.

Studies of clustering for non-leukemia CC have been conducted in different continents showing some heterogeneity in their results. In Europe, Ortega *et al*. ^19^ used elliptic analysis to identify clusters of CC in children under 15 years of age in Murcia, Spain between 1998 and 2009. This analysis identified a space-time cluster of lymphomas between 2011 and 2013. Also in Spain, a spatial case-control analysis between 1985 and 2015 including data from five autonomous regions explored the clustering of non-leukemia CC by site of residence and date of diagnosis; the authors found spatial clusters for all CC combined and for lymphomas at date of diagnosis, and for CNS embryonal tumors clustering at birth and diagnosis. The results, however, did not reach statistical significance for evidence of clustering when adjusted for multiple testing ^5^.

In the Asian continent, a study in Palestine performed an analysis of CC clusters between 1998 and 2007 using the circular scan method; a greater clustering effect was found in metropolitan districts and one cluster of lymphomas was identified in an agricultural city between 1998 and 2002 ^20^. In North America, Torabi and Rosychuk explored the presence of clusters of CC between 1983 and 2004 in the province of Alberta, Canada, using five different methods to analyze clustering, including circular scan tests. The study showed evidence of clustering to the south of the province but did not showed results by type of cancer ^21^. In South America, in the province of Cordoba, Argentina, Agost reported one of the first studies in the region using the circular scan test to detect clusters of CC. Spatial clusters were found for leukemias, lymphoid neoplasms, CNS tumors and in the space-time analysis clusters of neuroblastoma and other peripheral tumors were also identified ^22^.

Overall, the European studies tend to report lack of evidence for CC clusters, whereas other continents (such as in this study) tend to show some clustering evidence. The heterogeneous nature of the findings could be related to different factors, primarily environmental conditions and the methods used. In classic epidemiology, the consistency of the results of association between exposure and events is core when assessing causality ^23^. Nonetheless, in the spatial analysis the focus is on the description of the patterns and not the causality; this is why the heterogeneity of the results is important in these exploratory studies, since it can reflect conditions or exposures that may vary between and within populations.

The results of the studies can also differ due to the diversity of methods used. The spatial studies based on the analysis of areas (ecological approach) such as this study, and the studies in Canada and in Palestine ^20– 22^, seem to be more sensitive to the detection of clusters compared to the results of the studies based on point analysis (case-control studies) conducted in Europe ^5, 13^. We used an ecological approach for this first exploratory study because of the quality of information available in the country at municipality level and the absence of official data sources for selecting comparable controls. Kulldorf’s circular scan tests was chosen because it is optimal to detect clusters in a regular way, it has excellent performance detecting rare diseases in large populations such as CC ^24^, and for its easy use through specific software that makes it standardized and reproducible.

Non-leukemia CC clusters identified in Colombia are located mainly in the central region of the country near the mountain ranges that blend with large zones of agriculture and mining. These combined zones can generate special environments that allow the interaction of infectious agents, environmental, and occupational conditions that may have a space and time effect in the incidence of events such as CC. There is evidence that exposure to arsenic ^25^ and pesticides ^26– 28^ is related to a greater risk of developing CC, especially leukemias, lymphomas and CNS tumors.

One of the main strengths of the study is the use of available information in the SIVIGILA that counts with nationwide coverage, a high-quality follow-up process, and the permanent adjustment of data by the National Health Institute. The inclusion of other types of CC, different to leukemia, in SIVIGILA was done in 2013; this is the reason we excluded this year from the study, in order to avoid bias in the reports during the transition period. Despite its systematic processes, SIVIGILA does not correspond to a specific population-based cancer registry, which operates in four Colombian cities ^29^. For this reason, we recognize as a limitation that in the SNCCC could exist some level of sub-registry caused by the limitation of the access to the health care services, especially in rural and isolated areas. Additionally, the limited number of reported cases for group IV and subsequent groups of the ICCC-3 did not allow for the analysis of other groups different to group III (CNS).

## Conclusion

The spatial distribution of non-leukemia CC seem to have clustered patterns in some regions of the country that suggest possible infectious, environmental or occupational factors related to its incidence. Future studies should assess the effect of these factors related to non-leukemia CC. 

## Data availability

The data referenced by this article are under copyright with the following copyright statement: Copyright: ï¿½ 2021 Manrique-Hernández EF et al.

Data associated with the article are available under the terms of the Creative Commons Attribution Licence, which permits unrestricted use, distribution, and reproduction in any medium, provided the original data is properly cited. 

### Source data

We declare that we have permission for the free use of this data.

Zenodo: Clustering of childhood cancer in Colombia: a nationwide study. http://doi.org/10.5281/zenodo.4488080 ^10^.

This project contains the following source data:

- Database SIVIGILA by municipality (database of cases by type of cancer and municipality (geographic location) taken from SIVIGILA data)

- Database DANE population (database of populations by municipality (geographic location) taken from DANE data)

Data are available under the terms of the Creative Commons Attribution 4.0 International license (CC-BY 4.0).
