# Clustering of non-leukemia childhood cancer in Colombia: a nationwide study

**DOI:** 10.12688/f1000research.27766.2

**Published:** 2021-06-25

**Authors:** Edgar F. Manrique-Hernández, Marcela Pilar Rojas Díaz, Laura Andrea Rodriguez-Villamizar

**Affiliations:** 1 Public Health Department, School of Medicine, Universidad Industrial de Santander. Bucaramanga, Santander, Colombia; 2 Instituto Nacional de Salud, Bogotá D.C., Colombia

**Keywords:** Cluster Analysis, Neoplasms, Childhood, Epidemiology, Colombia

## Abstract

**Background:** Childhood cancer is considered one the most important causes of death in children and adolescents, despite having a low incidence in this population. Spatial analysis has been previously used for the study of childhood cancer to study the geographical distribution of leukemias. This study aimed to identify the presence of space-time clusters of childhood of cancer excluding leukemia in Colombia between 2014 and 2017.

**Methods:** All incident cancer cases (excluding leukemia) in children under the age of 15 years that had been confirmed by the National Surveillance System of Childhood Cancer between 2014 and 2017 were included. Kulldorf’s circular scan test was used to identify clusters using the municipality of residence as the spatial unit of analysis and the year of diagnosis as the temporal unit of analysis. A sensitivity analysis was conducted with different upper limit parameters for the at-risk population in the clusters.

**Results**: A total of 2006 cases of non-leukemia childhood cancer were analyzed, distributed in 432 out of 1,122 municipalities with a mean annual incidence rate of 44 cases per million children under the age of 15. Central nervous system (CNS) tumors were the most frequent type. Two space-time clusters were identified in the central and southwest regions of the country. In the analysis for CNS tumors, a spatial cluster was identified in the central region of the country.

**Conclusions: **The distribution of non-leukemia childhood cancer seems to have a clustered distribution in some Colombian regions that may suggest infectious or environmental factors associated with its incidence although heterogeneity in access to diagnosis cannot be discarded.

## Introduction

Childhood cancer (CC) is considered one the most important causes of death in children and adolescents, despite having a low incidence. The World Health Organization (WHO) estimates that nearly 400,000 new cases of CC are diagnosed every year in children between 0 and 19 years of age ^1^. The mean annual worldwide incidence of CC was estimated at 140.6 cases per million children between the age 0–14 years in the period of 2001 to 2010 ^1^. In the Americas it has been estimated that every year there are approximately 27,000 new cases of cancer in children under the age of 14 years, with an estimated mortality rate of 10,000 deaths/year ^2^. The majority of the incident cases in the Americas belong to the Latin American and Caribbean region making up nearly 65% of the diagnosed cases ^2^.

CC is a set of diseases that does not have a clear etiology yet. There are several conditions that have been identified as risk factors which include genetic (some genetic syndromes and polymorphisms) and non-genetic factors being high-dose radiation, prior chemotherapy, and some viruses the most consistent in literature ^3^. Other potential non-genetic risk factors include, some infectious diseases, exposure to pesticides, benzene and radiation, alcohol consumption during pregnancy, smoking, and the socioeconomic condition of the family ^4– 6^. Some of these factors are more specific than others, as was found with Burkitt´s and Hodgkin´s lymphoma, where the Epstein-Barr virus plays a relevant role. However, there are still controversies surrounding the etiology of these diseases ^5^.

Spatial analysis allows the identification of geographical patterns of health and disease related events that point out variations between populations contributing to the generation of hypotheses about possible etiologies ^7^. Spatial analysis has been previously used for the study of CC, mainly for studying the geographical distribution of leukemias ^4, 8^, This type of analysis allows for the identification of space and time variations in a geographical area and clustering detection ^4^. Clusters of acute childhood leukemia have been identified in Colombia ^9^, but analyses for CC other than leukemia are scarce ^5, 10, 11^. The objective of this study was to perform an exploratory study with space-time aggrupation to identify clusters of incident cases of non-leukemia CC other than leukemia in Colombian municipalities between 2014 and 2017.

## Methods

### Population

Colombia is a country located in the north corner of South The Colombian population for 2018 was approximately 48 million people ^12^. Women make up 51.2%, and children under 15 years represent 22.5% (12% female and 11.5% male) of the total population. Most of the Colombian population live in urban areas (77.1%) ^12^.

### Cancer and population data sources

All incident cases of non-leukemia CC diagnosed in children under 15 years of age between 2014 and 2017 were included. The data source was the National Surveillance System for Public Health (SIVIGILA, for its name in Spanish), which registers the newly confirmed and probable cases of CC in a systematic and mandatory manner. All cases included in the study were confirmed cases. Surveillance for CC started in Colombia in 2008 with the registry of childhood leukemia cases and starting in 2013 the system registers all types of CC ^13^. SIVIGILA verifies the confirmation of reported cases according to the results of diagnostic tests such as myelograms, immunotyping, histopathology or cytogenetic tests; adjusting the real number of confirmed cases and the diagnosis date. De-identified non-leukemia CC data were provided by the National Health Institute (INS for its name in Spanish), allowing access to the following variables: municipality of residence, date of birth, diagnosis date and type of CC according to the International Classification of Childhood Cancer, Third Edition (ICCC-3) ^14^. Cases were assigned a consecutive number which cannot be used to identify cases. SIVIGILA is the most complete registry of CC in Colombia, taking into account that it has a nationwide coverage and the reports are updated weekly ^9^.

Data from CNS and miscellaneous intracranial and intraspinal neoplasms (Group III) cases according to the ICCC-3 ^14 ^was extracted for a sub-analysis. CNS tumors include malignant and non-malignant cases. This group is the second with the highest incidence after leukemias ^5, 15^.

Data for the at-risk population in the 1122 municipalities of Colombia was provided by the National Department of Statistics (DANE for its name in Spanish) ^10^ which performed its last national census in 2018. For the calculation of the population between the years 2014 and 2017 the dynamics of DANE projections of population was used, and an interpolation of the population was conducted for each one of the municipalities for previous years ^16^. The childhood population under 15 years varies widely across municipalities with a mid-period population with a mean of 9,880 children, median of 3,336. The minimum childhood populations is located in La Guadalupe municipality of Guainía (149 children) and maximum in Bogotá, the capital district (1,381,081 children). The calculation of the coordinates (longitude and latitude) of the centroid of each municipality was done in QGIS version 3.16.3 using free cartographic information from the DANE Geoportal ^17^.

### Statistical analysis

We performed a descriptive analysis calculating frequencies with percentages and incidence rates. The incidence of CC was calculated for each municipality and a direct standardization by age and sex of the incidence rates was conducted using as reference the structure of children population for Colombia in 2017. Standardized rates and their respective confidence interval were calculated in STATA® version 14. The global Moran index was calculated to estimate the spatial autocorrelation. The analysis considered neighboring based on the distance between the municipality´s centroids calculated as the Euclidean distance measured between two centroids of municipalities (with no threshold specification). Choroplethic maps were built in order to visualize the standardized rates using the WGS84 projection for Colombia and the cartographic archives available for each municipality in the DANE cartography website ^17^. Moran´s index and maps were obtained site using ArcGIS version 10.3 and QGIS version 3.16.3.

Kuldorff’s circular scan test was used to identify spatial and spatio-temporal clusters ^18^, using the SaTScan® software version 9.6. This is a spatial hypothesis test that runs consecutive scans in the study area with different circumference radii that increase in size; the null hypothesis of the test is that the risk of the event (in this case risk of non-leukemia CC) within the circle is the same as outside the scanned area. Space and space-time exploratory analysis were run using a Poisson distribution and scanning for high rates; the space analysis unit was the municipality of residence and the time analysis unit was the year of diagnosis. The selection of the most likely cluster was selected based on the p-value of the log likelihood ratio (p>0,05 was considered statistically significant) and 999 replications were used in the simulation to evaluate the significance of the inference. We used an upper limit of the population at risk of 25% and for a sensitivity analysis we assess the results using upper limits of 50% and 10% to identify consistency of clustering results across different upper limits. We used Kulldorff’s spatio-temporal scan statistics because it is commonly used to detect spatial and/or temporal disease clusters in epidemiological studies and are appropriated for detecting regularly shaped clusters which we expect to find if clusters are related to localized environmental exposures at municipality level; this method have very good performance to detect large compact clusters of rare diseases in large territories compared to other scan methods ^19^, and it has a open software to implement the analysis which make it highly reproducible ^20^. As part of the sensitive analysis, we also used the Besag and Newell´s (BN) statistic as an additional method for assessing spatial clustering which tests each geographic area separately and combines them to obtain a specific cluster size (k) ^21^. We ran the BN statistic with different cluster sizes (k=10,20,30,50) using the DCluster package in R software.

### Ethical approval

This research received ethical approval from the ethics committee of scientific research at the Universidad Industrial de Santander (CEINCI UIS), on October 27, 2017 (approval number 24-2017).

## Results

### Study population

**Figure 1.**
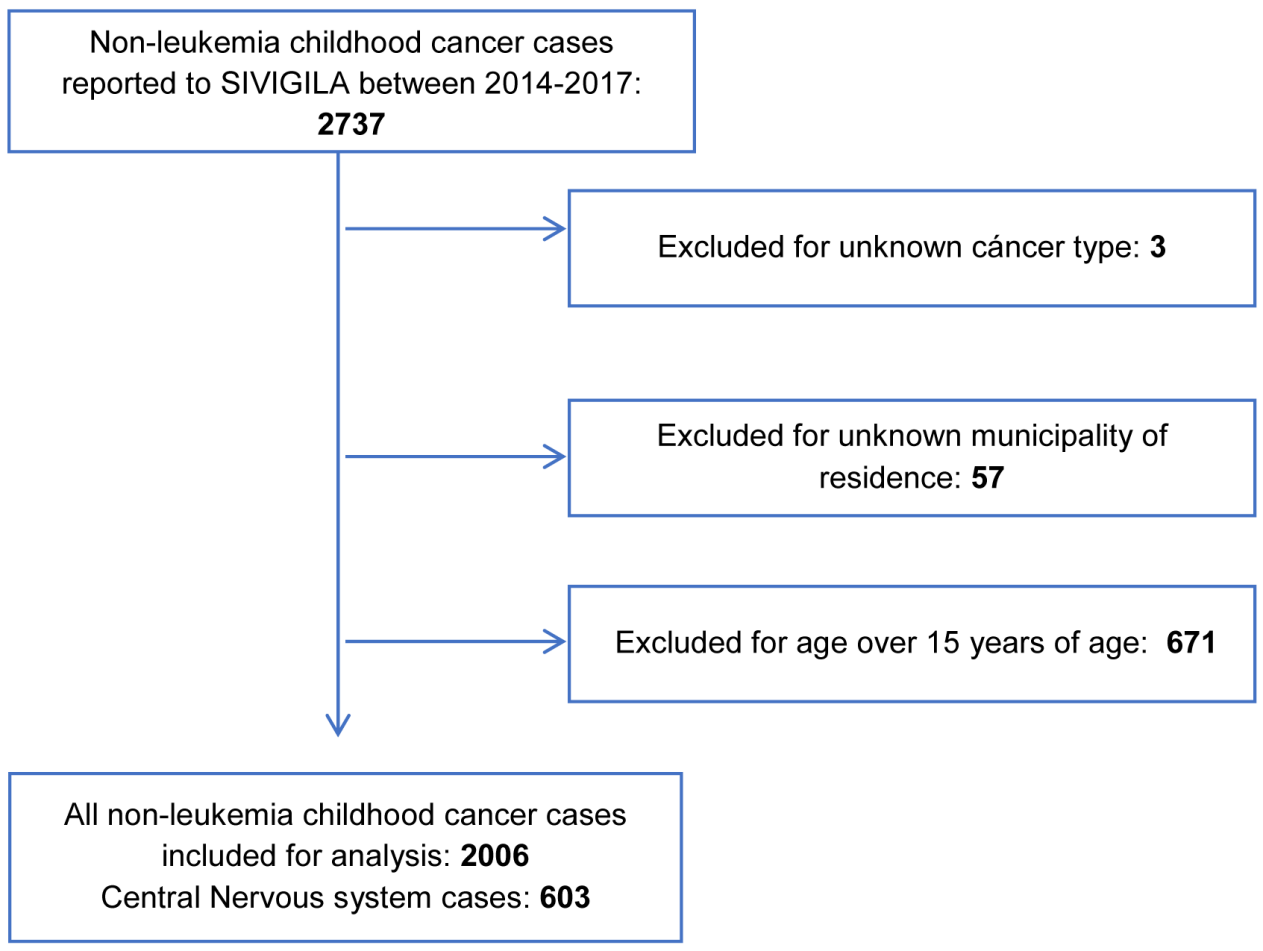
Study population selection flow chart.

SIVIGILA reported 2737 cases of non-leukemia CC between January 1st 2014 and December 31st 2017. A total of 731 cases were excluded for different reasons ( Figure 1). A total of 57 cases were reported with codification for department with no specification of municipality. The 57 cases belonged to 20 departments distributed across the country (Atlántico, Magdalena, Meta, Cesar, La Guajira, Valle, Tolima, Antioquia, Cundinamarca, Huila, Caquetá, Casanare, Amazonas, Chocó, Putumayo, Cauca, Santander, Bolívar, Norte de Santander y Córdoba). Therefore, a total of 2006 cases were included for the analysis, which were reported in 432 out of the 1122 municipalities of Colombia (38.5%). The analysis of CNS tumors included 603 cases reported in 201 municipalities (17.9%). The distribution of cases by sex, age group, year of diagnosis, and department of residence are presented in Table 1.

**Table 1.** Characteristics of the study population.

	All non-leukemia childhood cancer (n=2006)			Central nervous system tumors (n=603)		
Variable	No. Cases	%	Cumulative %	No. Cases	%	Cumulative %
**Sex**						
Female	908	45.26	45.26	269	44.61	44.61
Male	1.098	54.74	100.00	334	55.39	100.00
**Age (years)**						
0 – 4 years	783	39.03	39.03	207	34.33	34.33
5 –9 years	663	33.05	72.08	238	39.47	73.80
10–14 years	560	27.92	100.00	158	26.20	100.00
**Year of** **diagnosis**	**No. Cases**	**%**	**IR per million**	**No. Cases**	**%**	**IR per million**
2014	283	14.11	25.52	78	12.94	7.03
2015	544	27.12	48.54	179	29.68	15.97
2016	590	29.41	52.06	165	27.36	14.56
2017	589	29.36	51.39	181	30.02	15.79
**Department**	**No. Cases**	**%**	**MIR per million**	**No. Cases**	**%**	**MIR per million**
Amazonas	2	0.10	69.58	1	0.17	34.79
Antioquia	206	10.27	149.95	62	10.28	45.13
Arauca	11	0.55	160.58	3	0.50	43.80
Atlántico	26	1,35	42.72	5	0.83	8.21
Bogotá. D.C.	423	21.09	299.81	148	24.54	104.90
Bolívar	111	5.53	202.30	26	4.31	47.39
Boyacá	53	2.64	187.07	17	2.82	60.00
Caldas	56	2.79	288.44	14	2.32	72.11
Caquetá	21	1.05	168.63	5	0.83	40.15
Casanare	22	1.10	192.40	6	1.00	52.47
Cauca	57	2.84	153.98	17	2.82	45.92
Cesar	23	1.15	66.85	10	1.66	29.07
Choco	10	0.50	53.96	1	0.17	5.40
Cordoba	23	1.15	47.88	7	1.16	14.57
Cundinamarca	175	8.72	290.14	54	8.96	89.53
Guainía	3	0.15	147.90	2	0.33	98.60
Guajira	14	0.70	48.58	3	0.50	10.41
Guaviare	4	0.20	151.56	0	0	0.00
Huila	45	2.24	146.86	8	1.33	26.11
Magdalena	22	1.10	57.92	6	1.00	15.80
Meta	61	3.04	226.23	27	4.48	100.14
Nariño	56	2.79	142.00	17	2.82	43.11
Norte Santander	37	1.84	105.31	9	1.49	25.61
Putumayo	15	0.75	150.58	5	0.83	50.19
Quindío	23	1.15	236.91	5	0.83	51.50
Risaralda	32	1.60	167.41	10	1.66	52.32
Santander	140	6.98	295.97	38	6.30	80.34
Sucre	23	1.15	95.77	3	0.50	12.49
Tolima	63	3.14	203.36	15	2.49	48.42
Valle del Cauca	243	12.11	227.73	79	13.10	74.04
Vaupés	3	0.15	157.96	0	0	0.00
Vichada	2	0.10	48.09	0	0	0.00

IR: Incidence Rate; MIR: Mean annual Incidence Rate 2014–2017

**Figure 2.**
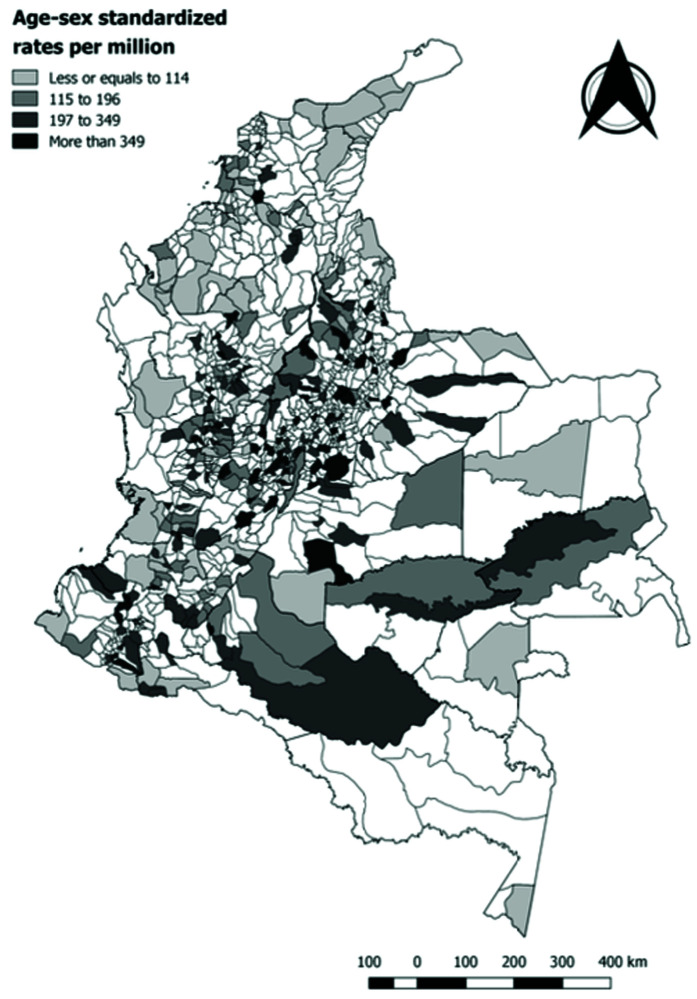
Standardized rates of non-leukemia childhood cancer by municipality, Colombia 2014–2017.

A slight majority of reported cases corresponded to males (54.74%) and 39.03% were reported in children under five years of age (0–4 years 33.5%, 5–9 years 36.99%, 10–14 years 29.51%). The mean annual incidence rate of non-leukemia CC was of 44 cases per million children under 15 years of age between 2014 and 2017 in Colombia. The highest incidence rates were reported in Meta (Villavicencio), Bogota D.C., Santander (Bucaramanga, Floridablanca), Bolivar (Cartagena), Valle del Cauca (Cali), Antioquia (Medellin), Cundinamarca (Soacha), Nariño (Pasto). The standardized rates by age and sex varied between 0 and 198 cases per million inhabitants under 15 years of age ( Figure 2). The Moran index was of 0.0023 (p=0.211) which indicates a low spatial autocorrelation of the incidence rates across Colombian municipalities.

For CNS tumors, again the slight majority of cases were reported in the male population (55.39%) and the 39.47% of the cases were reported in children between five and nine years of age. The departments with the highest number of cases were Bogota D.C, Valle del Cauca (Cali and Palmira), Antioquia (Medellin), Bolivar (Cartagena), Meta (Villavicencio), Santander (Bucaramanga), Cundinamarca (Soacha) and Nariño (Pasto).

### Clustering results

We identified four clusters in the spatial analysis for non-leukemia CC with overlap in the three clusters located in the center of the country which made up a large single cluster. The first cluster in the central region of the country included 327 municipalities distributed in the following departments: Cundinamarca (95), Meta (6), Boyaca (122), Santander (76), Antioquia (7), Caldas (4), Casanare (13), Tolima (3) and Bogota D.C

This cluster has a radius of 172.11 Km and 798 cases, with an expected number of cases of 497.88 with a relative risk (RR)=2 and p value <0.0001. The second cluster was identified in the departments of Cundinamarca (57), Meta (7) and Bogota D.C. with a radius of 72.04 km, and superposition with the first cluster; in this cluster 623 cases were identified for a total of 358.29 expected cases with RR = 2.07 and p value <0.0001. The third cluster was identified in the southwest region of Colombia, corresponding to the city of Cali (Valle del Cauca) and without superposition with any other cluster. In this third cluster 152 cases were identified where 87.95 cases were expected, with a RR = 1.79 and a p value <0.0001. The fourth cluster was identified in 23 municipalities of the Santander (Northeast region) department, with a 58.50 km radius, super positioning with some municipalities from the first cluster; in this cluster there were 106 cases with 55.15 as the expected number of cases, obtaining a RR = 1.97 and a p value <0.0001. When conducting the clustering analysis with no overlap we confirm the presence of two spatial clusters being the first and larger located at the center of the country and the second in the southwest region.

The same two clusters were identified in the space-time analysis for non-leukemia CC. The first cluster was located in the central region of the country corresponding to the following departments: Boyaca (122), Santander (76), Cundinamarca (95), Casanare (13), Meta (6), Caldas (4), Antioquia (7), Tolima (3), Bogota D.C. This cluster was identified between 2015 and 2016 with a radius of 172.11 Km, 491 cases reported with 249 expected obtaining a RR = 2.29 and p value <0.0001. The second cluster was identified in the city of Cali between 2016 and 2017, with 97 cases reported and 43.7 expected obtaining a RR = 2.28 and p value <0.0001.

The spatial analysis for CNS tumors identified one cluster located in the center of the country in the following departments: Meta (27), Cundinamarca (86), Casanare (8), Huila (1), Tolima (8) and Bogota D.C. This cluster has a radius of 177.43 km, with 237 cases reported and 122 expected for a RR = 2.55 and a p value <0.0001. The space-time analysis for CNS identified the same cluster in the central region corresponding to the following municipalities: Meta (16), Cundinamarca (95), Boyaca (30), Casanare (3), Tolima (1) and Bogota D.C. This cluster was identified between 2015 and 2016 with a radius of 112.53 km, with 143 cases reported and 58.9 expected obtaining a RR = 2.87 and p values <0.0001

**Figure 3.**
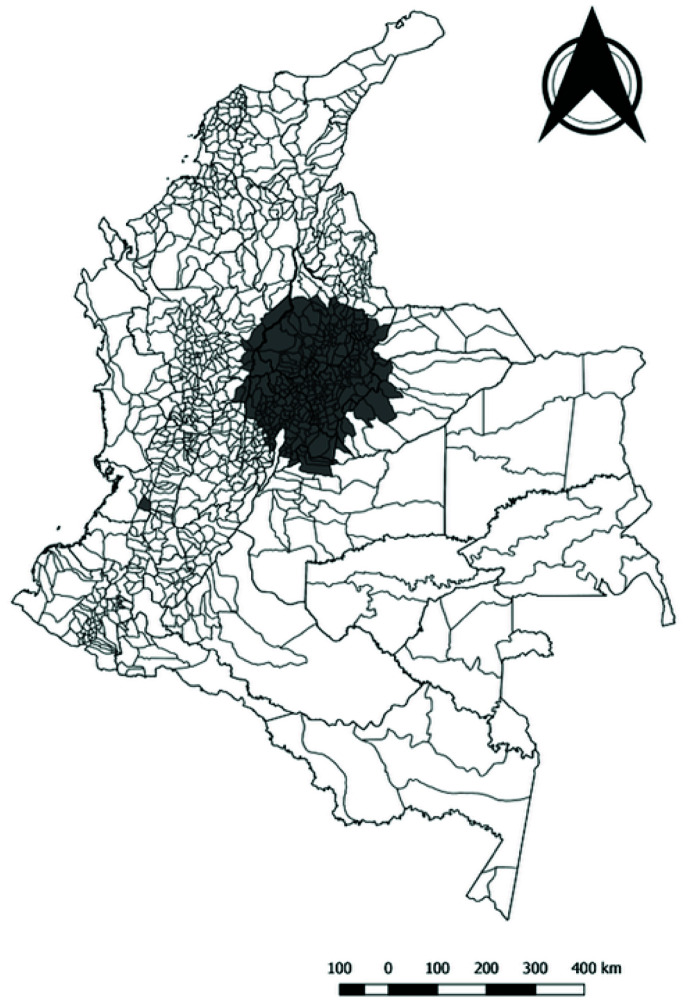
Municipalities consistently identified within spatial clusters of non-leukemia childhood cancer in Colombia, 2014–2017.

In the sensitivity analysis for non-leukemia CC circular scan tests were run using values of the at-risk population of 10% and 50%. There were 304 identified municipalities in the central region of the country that showed consistency in the three analysis (using 10%, 25% and 50% upper limit of at-risk population) and represent the two clusters identified in the center and southwest regions of Colombia ( Figure 3). The sensitivity analysis of spatial clustering results using the Besag-Newell statistic with k=30 also identified three clusters of municipalities located predominantly in the center of the country and one at the southwest of the country. The test was statistically significant for 18, 61 and 98 municipalities for cluster sizes of 10, 20, and 30 cases, respectively.

## Discussion

This study identified the presence of non-leukemia CC clusters between 2014 and 2017 in Colombia, using information with nationwide coverage available in SIVIGILA. To our knowledge, this is the first nation-wide study in South America using spatial analysis to describe the distribution and clustering of non-leukemia CC.

Spatial and spatio-temporal analysis have been previously used in CC, mainly in the study of the geographic pattern of leukemias ^8, 22^. A recent systematic review of space-time analysis identified 70 studies published up to 2016 of which 47 reported results for leukemias, 26 for lymphomas, 13 for CNS tumors and 12 for other types of tumors ^23^. All 32 analyses used for the meta-analysis were from Europe and United States; and the analysis showed evidence of leukemia clustering in children between 0 and 5 years of age. However, the evidence was not conclusive for lymphomas and CNS tumors. This study, however focuses on space-time clustering not on cluster detection which is aimed to detect localized excesses of CC cases.

Studies of clustering for non-leukemia CC have been conducted in different continents showing some heterogeneity in their results. In Europe, Ortega *et al.* ^24^ used elliptic analysis to identify clusters of CC in children under 15 years of age in Murcia, Spain between 1998 and 2009. This analysis identified a space-time cluster of lymphomas between 2011 and 2013. Also in Spain, a spatial case-control analysis conducted between 1985 and 2015 including data from five autonomous regions explored the clustering of non-leukemia CC by site of residence and date of diagnosis. The authors found spatial clusters for all CC combined and for lymphomas at date of diagnosis, and for CNS embryonal tumors clustering at birth and diagnosis. The results, however, did not reach statistical significance for evidence of clustering when adjusted for multiple testing ^5^. In France, a study of clustering of CC between 2000 and 2014 used different spatial scan methods and different geographical scales and found spatial heterogeneity and two large clusters for SNC tumors (glioma) and non-Hodgkin lymphoma ^11^.

In the Asian continent, a study in Palestine performed an analysis of CC clusters between 1998 and 2007 using the circular scan method; a greater clustering effect was found in metropolitan districts and one cluster of lymphomas was identified in an agricultural city between 1998 and 2002 ^25^. In Canada, Torabi and Rosychuk explored the presence of clusters of CC between 1983 and 2004 in the province of Alberta, Canada, using five different methods to analyze clustering, including circular scan tests. The study showed evidence of clustering to the south of the province but did not showed results by type of cancer ^26^. Then, a specific analysis of leukemia and lymphoma did not find specific spatiotemporal clustering ^27^. In Florida, United States, a study assessed the clustering of CC (0–19 years) between 2000–2010 using spatial scan methods applied to zip code areas and found evidence of clustering for CNS, leukemia and lymphoma ^10^. In South America, in the province of Cordoba, Argentina, Agost reported one of the first studies in the region using the circular scan test to detect clusters of CC. Spatial clusters were found for leukemias, lymphoid neoplasms, CNS tumors and in the space-time analysis clusters of neuroblastoma and other peripheral tumors were also identified ^28^.

Overall, most European studies tend to report lack of evidence for CC clusters, whereas other continents (such as in this study) tend to show some clustering evidence. The heterogeneous nature of the findings could be related to different factors, primarily environmental conditions and the methods used. In classic epidemiology, the consistency of the results of association between exposure and events is core when assessing causality ^29^. Nonetheless, in the spatial analysis the focus is on the description of the patterns and not the causality; this is why the heterogeneity of the results is important in these exploratory studies, since it can reflect conditions or exposures that may vary between and within populations.

The results of the studies can also differ due to the diversity of methods used. The spatial studies based on the analysis of areas (ecological approach) such as this study, and the studies in Argentina, Canada, France and in Palestine ^11, 19, 25, 26, 28^, seem to identify more often clusters compared to the results of the studies based on point analysis (case-control studies) conducted in Europe ^5, 15^. However, differences might be explained for difference in distribution of CC cases in countries. We used an ecological approach for this first exploratory study because of the quality of information available in the country at municipality level and the absence of official data sources for selecting comparable controls. Kulldorf’s circular scan tests was chosen because it is optimal to detect clusters in a regular way, it has excellent performance detecting rare diseases in large populations such as CC ^19^, and for its easy use through specific software that makes it standardized and reproducible.

Non-leukemia CC clusters identified in Colombia are located mainly in the central region of the country. One cluster for childhood leukemia was also identified in the center of the country in a previous study ^9^. Clusters for both leukemia and non-leukemia cases might be related to each other, however the non-leukemia cluster is larger (327 municipalities compared with 109 identified in the leukemia cluster), more expanded to the North of the leukemia cluster, and with higher incidence rates located in municipalities with predominant rural areas. The cluster location corresponds to a large area in the mountain ranges that blend with large zones of agriculture and mining operations. These combined zones can generate special environments that allow the interaction of infectious agents, environmental, and occupational conditions that may have a space and time effect in the incidence of events such as CC. There is evidence that exposure to arsenic ^30^ and pesticides ^31– 33^ is related to a greater risk of developing CC, especially leukemias, lymphomas and CNS tumors. The large area covered by the central cluster and its high relative risk is of concern and suggest the presence of an infectious or environmental factor strongly associated to the risk of CC, mainly non-leukemia, that is highly prevalent in this area compared to the remaining areas of the country. Further studies using ecological and individual approaches should be conducted addressing the relationship of CC cases with specific infectious, environmental, and occupational exposures in Colombia.

The spatial heterogeneity in this type of ecological spatial analysis can be also observed due to diagnosis or reporting heterogeneity. For this study we selected as source of cancer cases the report to the national surveillance system for childhood cancer (NSSCC) from national public health surveillance system (SIVIGILA) because this is the strongest and more complete health information system that is operating in all 1,122 municipalities in Colombia. Unfortunately, the cancer population-based registries in Colombia are limited to four regions in the country which are representative of specific urban areas but do not represent the full spectrum of municipalities and regions in Colombia ^34^. The SIVIGILA is operated by the National Institute of Health (INS for Spanish) as a mandatory, systematic, and continuous registry with standardized protocols. The system operates permanently in all municipalities based on immediate report for selected health events and weekly report for all events, including childhood cancer. The CC surveillance began in 2008 when acute leukemia was included as a mandatory health notification event. In 2013 the system was extended to all types of childhood cancer. The system preserved the core formats and software for reporting acute childhood leukemia and therefore the extension to other cancer types had a shorter learning curve for the surveillance system´s personnel in municipalities. During the study period, notification of non-leukemia cancer were reported for 432 municipalities in almost all departments and districts (including municipalities with predominantly rural remote areas), which support the wide coverage of the surveillance system.

For a previous study, we conducted a comparison between national high-cost account registry and SIVIGILA report during 2016. We identified 1394 incident cases of CC and 1206 (86.5%) of them were reported to SIVIGILA, indicating that the systems captured 83% of all incident cases of CC in Colombia, which included non-leukemia cases ^9^. The 188 cases missing in SIVIGILA corresponded to different cancer diagnosis and municipalities distributed in 28 departments across the country. Therefore, we assumed that the presence of underreporting it is not concentrated in specific areas of the country and underreporting although is present, might not be the main explanation for the spatial heterogeneity in our results. However, health care access to cancer diagnosis is limited to specific regions in the country located mainly in the main capital cities and therefore delay in diagnosis (and derived delayed in reporting) might be present in remote semirural and rural municipalities ^35^. This study analyzed CC data for 2014–2017 and databases were consolidated in 2019, therefore cases with delayed diagnosis had the opportunity to be included in the last two years as SIVIGILA required the reporting of incident and prevalent cases since 2014 for non-leukemia cases. However, the cases with missing diagnosis due to limitations in access to health care might be still present in the study but cannot be quantified.

One limitation of this study is that the cluster frontier for the main central cluster was difficult to delineate in the overlapping analysis. This is a known limitation of the cluster detection methods and for the Kulldorf´s scan test used it is added to its limitation to detect clusters with irregular shapes. We conducted a sensitivity analysis with different population at-risk proportions and to delineate better the cluster and Besag-Newell statistic as alternative spatial clustering method with similar results, however further studies might include alternative clustering methods . Other important limitation of our study is that we assessed clusters based on place of residence at the time of diagnosis but we were not able to compare with clusters based on place of residence at birth or during gestation as this information was not available in SIVIGILA. Additionally, the limited number of reported cases for group IV and subsequent groups of the ICCC-3 did not allow for the analysis of other groups different to group III (CNS).

## Conclusion

The spatial distribution of non-leukemia CC seem to have clustered patterns in some regions of the country that suggest possible infectious, environmental or occupational factors related to its incidence. Future studies should assess the effect of these factors related to non-leukemia CC.

## Data availability

### Source data

We declare that we have permission for the free use of this data.

Zenodo: Clustering of childhood cancer in Colombia: a nationwide study. http://doi.org/10.5281/zenodo.4781513 ^36^.

This project contains the following source data:

- Database SIVIGILA by municipality (database of cases by type of cancer and municipality (geographic location) taken from SIVIGILA data)

- Database DANE population (database of populations by municipality (geographic location) taken from DANE data)

Data are available under the terms of the Creative Commons Attribution 4.0 International license (CC-BY 4.0).
