# Supplementary material for: Clustering of non-leukemia childhood cancer in Colombia: a nationwide study
Source: F1000Res. 2021 Jun 25;10:86. Originally published 2021 Feb 9. [Version 2] doi: 10.12688/f1000research.27766.2 (PMC8261763; doi:10.12688/f1000research.27766.2)
Supplement: Peer review history

# Peer review: Reviewer response for version 2

**DOI:** 10.5256/f1000research.57186.r88297

**Published:** 2021-07-22

**Authors:** Lisandro Agost

**Affiliations:** 1 Centro de Ecología y Recursos Naturales Renovables (CERNAR) – IIByT CONICET-UNC, Córdoba, Argentina

The authors have incorporated and responded to all suggestions satisfactorily. I believe the article is now ready for indexing.

Is the work clearly and accurately presented and does it cite the current literature?

Yes

If applicable, is the statistical analysis and its interpretation appropriate?

Yes

Are all the source data underlying the results available to ensure full reproducibility?

Partly

Is the study design appropriate and is the work technically sound?

Yes

Are the conclusions drawn adequately supported by the results?

Partly

Are sufficient details of methods and analysis provided to allow replication by others?

Partly

Reviewer Expertise:

Ecology - Epidemiology - Childhood Cancer - Pesticide Risk Indicators

I confirm that I have read this submission and believe that I have an appropriate level of expertise to confirm that it is of an acceptable scientific standard.

# Peer review: Reviewer response for version 2

**DOI:** 10.5256/f1000research.57186.r88298

**Published:** 2021-07-09

**Authors:** Smita Asthana

**Affiliations:** 1 Division of Epidemiology and Biostatistics, Institute of Cytology and Preventive Oncology, NOIDA, New Delhi, Delhi, India

All the suggestions are incorporated by the authors. Changes made are satisfactory and I recommend the article for indexing.

Is the work clearly and accurately presented and does it cite the current literature?

Yes

If applicable, is the statistical analysis and its interpretation appropriate?

Yes

Are all the source data underlying the results available to ensure full reproducibility?

Yes

Is the study design appropriate and is the work technically sound?

Yes

Are the conclusions drawn adequately supported by the results?

Yes

Are sufficient details of methods and analysis provided to allow replication by others?

Yes

Reviewer Expertise:

NA

I confirm that I have read this submission and believe that I have an appropriate level of expertise to confirm that it is of an acceptable scientific standard.

# Peer review: Reviewer response for version 2

**DOI:** 10.5256/f1000research.57186.r88296

**Published:** 2021-07-06

**Authors:** Richard J.Q. McNally

**Affiliations:** 1 Institute of Health and Society, Newcastle University, Newcastle, UK

I am happy with the revisions that have been made.

Is the work clearly and accurately presented and does it cite the current literature?

Partly

If applicable, is the statistical analysis and its interpretation appropriate?

Partly

Are all the source data underlying the results available to ensure full reproducibility?

Yes

Is the study design appropriate and is the work technically sound?

Partly

Are the conclusions drawn adequately supported by the results?

Partly

Are sufficient details of methods and analysis provided to allow replication by others?

Partly

Reviewer Expertise:

Epidemiology

I confirm that I have read this submission and believe that I have an appropriate level of expertise to confirm that it is of an acceptable scientific standard.

# Peer review: Reviewer response for version 1

**DOI:** 10.5256/f1000research.30704.r81746

**Published:** 2021-05-07

**Authors:** Richard J.Q. McNally

**Affiliations:** 1 Institute of Health and Society, Newcastle University, Newcastle, UK

This is an interesting paper reporting clustering of childhood cancer in Colombia. However, there are a number of specific issues and these are listed below:

1. There needs to be more justification for choice of methods used.
2. Is the interest only in the identification of specific spatial or space-time clusters, rather than more generalised spatial or space-time clustering?
3. The presence of specific clusters is more consistent with some localised environmental sources of exposure, rather than general exposures (such as infections). Could these clusters be linked with data on more specific localised exposures?
4. I suggest that the authors also consider other methods for looking at generalised spatial clustering of space-time clustering. These include methods of Cuzick, Besag, Knox, Jacquez and Diggle. 
5. The English language needs improving throughout.

Is the work clearly and accurately presented and does it cite the current literature?

Partly

If applicable, is the statistical analysis and its interpretation appropriate?

Partly

Are all the source data underlying the results available to ensure full reproducibility?

Yes

Is the study design appropriate and is the work technically sound?

Partly

Are the conclusions drawn adequately supported by the results?

Partly

Are sufficient details of methods and analysis provided to allow replication by others?

Partly

Reviewer Expertise:

Epidemiology

I confirm that I have read this submission and believe that I have an appropriate level of expertise to state that I do not consider it to be of an acceptable scientific standard, for reasons outlined above.

# Response

**Published:** 2021-05-24

**Authors:** Edgar F. Manrique-Hernández

**Affiliations:** Universidad Industrial de Santander, Colombia

Reviewer #4

**Richard J.Q. McNally**, Institute of Health and Society, Newcastle University, Newcastle, UK 

 This is an interesting paper reporting clustering of childhood cancer in Colombia.

**Author's response to reviewer:**

 Thank you for your comment.

 However, there are a number of specific issues and these are listed below:

  

1. There needs to be more justification for choice of methods used.

**Author's response to reviewer:**

 Thank you for your comment. We have added a comment on methods section for the selection of the circular scan test.

 We used Kulldorff’s spatio-temporal scan statistics because it is commonly used to detect spatial and/or temporal disease clusters in epidemiological studies and are appropriated for detecting regularly shaped clusters which we expect to find if clusters are related to localized environmental exposures at municipality level; this method have very good performance to detect large compact clusters of rare diseases in large territories compared to other scan methods, and it has a open software to implement the analysis which make it highly reproducible.

  

1. Is the interest only in the identification of specific spatial or space-time clusters, rather than more generalised spatial or space-time clustering?

**Author's response to reviewer: **Thank you for your comment. We were interested in overall clustering but specifically in the identification of localized spatial or space-time clusters. That is the reason we used a scan clustering method.

  

1. The presence of specific clusters is more consistent with some localised environmental sources of exposure, rather than general exposures (such as infections). Could these clusters be linked with data on more specific localised exposures?

**Author's response to reviewer: **Thank you for your comment. We conducted this exploratory study to assess the presence of localized clusters and as commented in the discussion sections, the localized central cluster open the door to further studies assessing specific localized exposures mainly related to environmental and occupational exposures related to pesticide’s uses and mining operations in the localized area identified.

  

1. I suggest that the authors also consider other methods for looking at generalised spatial clustering of space-time clustering. These include methods of Cuzick, Besag, Knox, Jacquez and Diggle. 

**Author's response to reviewer: **Thank you for your suggestion. We have added the Besag and Newell´s statistic as an additional method for assessing spatial clustering and we found similar results at different cluster sizes.

1. The English language needs improving throughout.

**Author's response to reviewer: **Thank you for your comment. We have reviewed and improved the language style.

# Peer review: Reviewer response for version 1

**DOI:** 10.5256/f1000research.30704.r81740

**Published:** 2021-04-21

**Authors:** Stéphanie Goujon

**Affiliations:** 1 Childhood and adolescent cancer team (EPICEA), UMR 1153 Centre of Research in Epidemiology and StatisticS (CRESS), Villejuif, France

The nationwide study conducted by Manrique-Hernández and colleagues described the space-time distribution of non-leukemia childhood cancers in Colombia. Based on data from the national health surveillance system, the study aimed at detecting spatial and spatio-temporal localised excesses of cases over the period 2014-2017, on the municipality scale. Describing spatial and temporal variations is of a great importance for childhood cancer surveillance. The authors used registry based data and the scan method developed by M. Kulldorff, which is appropriate for such a study. In a purely spatial analysis, they detected four widespread overlapping clusters of non-leukemia cases in two different areas, in which the number of observed cases were about twice the numbers expected under the hypothesis of homogeneous incidence rates over the whole study period. Space-time analyses identified clusters in the same areas, with excesses observed during shorter time periods.

**Major comments:**

- There is a major issue concerning the interpretation of the results.

The authors concluded that the detected clusters "may suggest infectious or environmental factors associated with its incidence" (abstract and main text).

 The presence of localized clusters might actually suggest that a risk factor is present with a higher prevalence in these places than elsewhere in Colombia, but spatial heterogeneity might also be due to differences in case registration. I consider this point is of a primary importance and should be discussed further in the paper and in the abstract (it was discussed rapidly at the end of the discussion section).

 To discuss that point, it would be useful to describe (at least briefly) in the paper the surveillance system in Colombia and to provide information on how the cases are identified nationwide. Are the data provided automatically to the NHS by hospital centers? Or do SIVIGILA members visit the hospital centers to collect data actively? Which hospital centers are visited or contacted? What are the main care pathways in Colombia? The authors cited several interesting papers that were written in Spanish and therefore not easily understandable.

 Spatial differences in case registration might be observed because of under-diagnoses or difficulties for the registry to identify cases in some regions. In the paper by Rodriguez-Villamizaron *et al. *on childhood leukemia (2020), the authors indicated that childhood cancer became a priority in Colombia since 2010. Do the authors consider that the new regulation was adopted homogeneously in Colombia since that date, or is it possible that access to diagnosis was different over the study period 2014-2017 depending on the place of residence? Besides, were all medical reports available nationwide during the study period?

 In Rodriguez-Villamizaron *et al.* 2020, under-registration was also discussed and it was estimated that 17% of AL cases were not captured by the registry in 2016 ("During this year [2016] 1394 incident cases of childhood cancer were identified and 1206 (86.5%) of them were reported to the NSSCC […] the NSSCC captured and registered 83% of all incident cases of childhood cancer in Colombia during 2016"). If the missing cases were not homogeneously distributed in the municipalities, this could lead to clusters in areas where registration was more exhaustive. Do the authors have similar information for non-leukemia cases?

 A large cluster of childhood leukemia was detected in the center of Colombia. Can it be related to the large cluster of non-leukemia cases reported in the present study?

- The four spatial clusters detected in the study were located in two distinct regions, as three clusters overlapped. The space-time clusters corresponded to the same spatial areas. In all, as a conclusion, I would say that only two clusters were detected (maybe over 2 years only) and I suggest to discuss the fact that the cluster frontiers were difficult to delineate (a well-known limit of the cluster detection methods). The size of the largest detected cluster, in the central region, and the magnitude o the relative risk should also be discussed. A relative risk of about 2 in such a large area is quite surprising and unexpected. If an infectious or environmental factor was responsible for such an excess, it would have to be strongly associated to the risk of non-leukemia cancer and highly prevalent in the cluster area (in comparison to the remaining part of Colombia).

- The statistical methods used in this study, Moran’s test for spatial autocorrelation and the Kulldorff’s scan method for cluster detection, were appropriate. However, some details on the methodology and the parameters used could be added. In particular, the authors could add a reference for the Moran’s test for spatial autocorrelation, and explain how the neighborhood was defined in the study. Were two municipalities considered neighboring areas if the distance between their centroids was below a given threshold or if they shared a common border?

It would also be useful to provide further details on the scan method (estimation of a likelihood ratio for each window and selection of the most likely cluster, i.e. the window associated to the maximum ratio), and the simulations that were conducted to evaluate the significance thresholds (how many simulations were done?).

  

- Several other studies should be referred to in the introduction and discussion sections to provide more accurate information on childhood cancer etiology and the literature on childhood cancer clusters.

(Suggested publications):

*Etiology*:

- 'Genetic and nongenetic risk factors for childhood cancer' by Spector *et al.* (2015 ^1^). 
- 'Environmental Exposure and Risk of Childhood Leukemia: An Overview' by Schüz *et al.* (2016 ^2^). 

*Clustering:*

- 'Childhood cancer trends in a western Canadian province: a population-based 22-year retrospective study' by Rosychuk *et al.* (2010 ^3^).
- 'Spatial clustering and space-time clusters of leukemia among children in Germany, 1987-2007' by Schmiedel *et al.* (2007 ^4^).
- 'Leukemia and lymphoma incidence in children in Alberta, Canada: a population-based 22-year retrospective study by Kulkarni *et al.* (2011 ^5^).
- 'A cluster analysis of Pediatric Cancer Incidence Rates in Florida: 2000-2010' by Amin *et al.* (2014 ^6^). 
- 'Spatial and temporal variations of childhood cancers: Literature review and contribution of the French national registry' by Goujon *et al.* (2018 ^7^).

**Minor comments:**

**Abstract:**

- "A sensitivity analysis was conducted with different upper limit parameters for the at-risk population." The upper limit was for the at-risk population included in the cluster.
- There are 1122 municipalities nationwide. Several municipalities had no observed cases, so that the cases were actually distributed in 432 different municipalities. However, all the municipalities were included in the analyses (if the at-risk population was not null). I suggest therefore to report that "2006 cases were distributed in 1122 municipalities".
- Regarding CNS tumors, it would be informative to specify whether non malignant cases were included.
- Conclusion: differences in case registration should also be considered as a possible explanation.

**Introduction:**

- Based on reference 1, "The mean annual incidence of CC was estimated at 140.6 cases per million children". This incidence rate was estimated worldwide, which could be specified.
- "There are several conditions that have been identified as risk factors". The factors cited in this sentence were associated with childhood cancer with different degree of evidence. Some factors are considered as known (high dose ionising radiation, chemotherapy, certain genetic syndromes and some genetic polymorphisms, some viruses in lymphomas) or highly suspected (domestic and occupational parental exposure, socioeconomic conditions, infections and immune system stimulation for leukemia, birth weight, benzene exposure, air pollution) risk factors, while for other factors the literature is more heterogeneous and no firm conclusion can be drawn to date (tobacco and alcohol consumption). It is important to consider that point when discussing the etiology.
- The following sentence is quite long and difficult to understand: "contributing to the generation of hypotheses about possible etiologies. Spatial analysis has been previously used for the study of CC, mainly for studying the geographical distribution of leukemias, since this type of analysis allows for the identification of space and time variations in a geographical area that generate clusters that indicate an increase in the tendency of the cases".
- At the end of the introduction, several studies on childhood cancer cluster detection are cited, but only one reference (ref 5) is provided for non-leukemia cancers. Maybe some of the references I reported above could be added here.

**Methods:**

- CC cases were identified by the National Surveillance System for Public Health, which registers "the newly confirmed and probable cases of CC". It is unclear to me what "probable cases" means? It seems that CC diagnoses are confirmed on the basis of diagnostic exams and coded according to the ICCC-3, so could the authors explain what are the probable cases that are registered (if not confirmed why are they registered?).
- Are non-malignant CNS tumors registered in SIVIGILIA, and included in the study? Based on the number of CNS tumor cases reported in the result section (17.9% of non-leukemia cases), I assume that only malignant CNS tumors were included in the study. This information should be added in the main text and the abstract.

**Statistical analysis:**

- "We performed a descriptive analysis calculating frequencies and central tendency measurements." I don’t understand what "central tendency measurements" refers to?
- I understand that standardized rates were considered to account for potential differences in the age distribution of the pediatric population between municipalities. Were those potential differences also accounted for in the SaTScan analyses?

**Results:**

- 731 cases were excluded from the analyses, of which 57 had an unknown municipality of residence. It would be interesting to describe those cases in terms of type of diagnosis, year of diagnosis. Could the authors get other geographic information related to the area of residence? Were those cases grouped in a particular region?
- It would be useful to describe the distribution of the pediatric population in the 1122 municipalities to illustrate the potential heterogeneity.
- The annual incidence rate for non leukemia cancer (44 cases/million) seems to be quite low compared to the overall incidence rate reported in Stealiarova-Foucher *et al. *2017 for South America (133.9 cases/million, table S3), even if leukemia were excluded, and compared to the range reported in figure 2 legend ("more than 349 cases/million in the highest category"). This point needs clarification.
- The scan method selects the most likely cluster on the basis of a likelihood ratio that is calculated for each spatial window (from one municipality to the maximum size defined by the user). Likelihood ratios are very similar between two consecutive windows (as only one municipality or a small number of municipalities is added to the window at each step) that’s why overlapping significant clusters can be detected (as in this study). Wouldn’t it be interesting to run the scan method to detect non-overlapping cluster (this is an option in SaTScan)?
- Figure 2 is not as clear as figure 5. The four clusters can’t be identified precisely. Presenting one map for each cluster may be useful (or just 3 overlapping circles around the detected cluster areas in the central area and another circle centered on Cali).

**Discussion:**

- The study cited in reference 18 focused on space-time clustering not on cluster detection. The authors reviewed the studies which tested for a space-time interaction, i.e. a general tendency of childhood cancer cases to occur more closely in space and time than expected under independent spatial and temporal patterns. This issue is really different from the question of detecting localized excesses of cases. The difference between space-time interaction and cluster detection (and even spatial clustering) should be clearly stated when presenting the results from reference 18.
- Regarding the heterogeneity of the previous study results on CC cluster detection, I agree with the authors that considering count data in geographical units or individual point data to detect localized clusters may lead to different conclusions, and may explained some differences between study results (end of page 7). However, I wouldn't say that the ecological approach is more sensitive than the point analysis on the basis of a small number of studies. 
  It may be that some excesses actually existed in some countries in some particular time periods (not necessarily related to an environmental factor), while cases were more homogeneously distributed in other countries. Sensitivity refers to situations of true excesses.
- At the end of the discussion, the authors noted "that in the SNCCC could exist some level of sub-registry caused by the limitation of the access to the health care services, especially in rural and isolated areas. "Again, this point is really important for the interpretation of the results (already noted as a major comment).

Is the work clearly and accurately presented and does it cite the current literature?

Partly

If applicable, is the statistical analysis and its interpretation appropriate?

Yes

Are all the source data underlying the results available to ensure full reproducibility?

Yes

Is the study design appropriate and is the work technically sound?

Yes

Are the conclusions drawn adequately supported by the results?

Partly

Are sufficient details of methods and analysis provided to allow replication by others?

No

Reviewer Expertise:

epidemiology; biostatistics; childhood cancer; space-time variations; environmental epidemiology;

I confirm that I have read this submission and believe that I have an appropriate level of expertise to confirm that it is of an acceptable scientific standard, however I have significant reservations, as outlined above.

## References

[1] : Genetic and nongenetic risk factors for childhood cancer. *Pediatr Clin North Am* .2015;62(1) : 10.1016/j.pcl.2014.09.013 11-25 10.1016/j.pcl.2014.09.013 25435109PMC4384439

[2] : Environmental Exposure and Risk of Childhood Leukemia: An Overview. *Arch Med Res* .47(8) : 10.1016/j.arcmed.2016.11.017 607-614 10.1016/j.arcmed.2016.11.017 28476188

[3] : Childhood cancer trends in a western Canadian province: a population-based 22-year retrospective study. *Pediatr Blood Cancer* .2010;55(7) : 10.1002/pbc.22785 1348-55 10.1002/pbc.22785 20830776

[4] : Spatial clustering and space-time clusters of leukemia among children in Germany, 1987-2007. *Eur J Epidemiol* .2010;25(9) : 10.1007/s10654-010-9488-7 627-33 10.1007/s10654-010-9488-7 20623321

[5] : Leukemia and lymphoma incidence in children in Alberta, Canada: a population-based 22-year retrospective study. *Pediatr Hematol Oncol* .2011;28(8) : 10.3109/08880018.2011.613091 649-60 10.3109/08880018.2011.613091 21981741

[6] : A Cluster Analysis of Pediatric Cancer Incidence Rates in Florida: 2000–2010. *Statistics and Public Policy* .2014;1(1) : 10.1080/2330443X.2014.928245 69-77 10.1080/2330443X.2014.928245

[7] : Spatial and temporal variations of childhood cancers: Literature review and contribution of the French national registry. *Cancer Med* .7(10) : 10.1002/cam4.1774 5299-5314 10.1002/cam4.1774 30230715PMC6198217

# Response

**Published:** 2021-05-24

**Authors:** Edgar F. Manrique-Hernández

**Affiliations:** Universidad Industrial de Santander, Colombia

Clustering of childhood cancer in Colombia: a nationwide study

**Reviewer #3**

**Stéphanie Goujon**, Childhood and adolescent cancer team (EPICEA), UMR 1153 Centre of Research in Epidemiology and StatisticS (CRESS), Villejuif, France 

*21 Apr 2021 | for Version 1*

 The nationwide study conducted by Manrique-Hernández and colleagues described the spacetime distribution of non-leukemia childhood cancers in Colombia. Based on data from the national health surveillance system, the study aimed at detecting spatial and spatio-temporal localised excesses of cases over the period 2014-2017, on the municipality scale. Describing spatial and temporal variations is of a great importance for childhood cancer surveillance. The authors used registry based data and the scan method developed by M. Kulldorff, which is appropriate for such a study. In a purely spatial analysis, they detected four widespread overlapping clusters of nonleukemia cases in two different areas, in which the number of observed cases were about twice the numbers expected under the hypothesis of homogeneous incidence rates over the whole study period. Space-time analyses identified clusters in the same areas, with excesses observed during shorter time periods.

Major comments:

 ○ There is a major issue concerning the interpretation of the results.

 The authors concluded that the detected clusters "may suggest infectious or environmental factors associated with its incidence" (abstract and main text). The presence of localized clusters might actually suggest that a risk factor is present with a higher prevalence in these places than elsewhere in Colombia, but spatial heterogeneity might also be due to differences in case registration. I consider this point is of a primary importance and should be discussed further in the paper and in the abstract (it was discussed rapidly at the end of the discussion section).

 To discuss that point, it would be useful to describe (at least briefly) in the paper the surveillance system in Colombia and to provide information on how the cases are identified nationwide. Are the data provided automatically to the NHS by hospital centers? Or do SIVIGILA members visit the hospital centers to collect data actively? Which hospital centers are visited or contacted? What are the main care pathways in Colombia? The authors cited several interesting papers that were written in Spanish and therefore not easily understandable.

 Spatial differences in case registration might be observed because of under-diagnoses or difficulties for the registry to identify cases in some regions. In the paper by RodriguezVillamizaron et al. on childhood leukemia (2020), the authors indicated that childhood cancer became a priority in Colombia since 2010. Do the authors consider that the new regulation was adopted homogeneously in Colombia since that date, or is it possible that access to diagnosis was different over the study period 2014-2017 depending on the place of residence? Besides, were all medical reports available nationwide during the study period?

 In Rodriguez-Villamizaron et al. 2020, under-registration was also discussed and it was estimated that 17% of AL cases were not captured by the registry in 2016 ("During this year [2016] 1394 incident cases of childhood cancer were identified and 1206 (86.5%) of them were reported to the NSSCC […] the NSSCC captured and registered 83% of all incident cases of childhood cancer in Colombia during 2016"). If the missing cases were not homogeneously distributed in the municipalities, this could lead to clusters in areas where registration was more exhaustive. Do the authors have similar information for non-leukemia cases?

**Author's response to reviewer:**

 Thank you for your detailed review and comments. Certainly, spatial heterogeneity in this type of ecological spatial analysis can be observed due to diagnosis or reporting heterogeneity.  For this study we selected as source of cancer cases the report to the national surveillance system for childhood cancer (NSSCC) from SIVIGILA because this is the strongest and more complete health information system that is operating in all 1,122 municipalities in Colombia. Unfortunately, the cancer population-based registries in Colombia are limited to four regions in the country which are representative of specific urban areas but do not represent the full spectrum of municipalities and regions in Colombia. The national surveillance system (SIVIGILA) is operated by the National Institute of Health (INS for Spanish) as a mandatory, systematic, and continuous registry with standardized protocols for more than 100 events of interest in public health. The system operates permanently in all municipalities based on immediate report for selected health events and weekly report for all events, including childhood cancer. The system is administrated and regulated by INS and operational support and training for municipalities is provided by the health secretary of each state (departments in Colombia). Childhood cancer surveillance began in 2008 when acute leukemia was included as a mandatory health notification event. In 2013 the system was extended to all types of childhood cancer. The system preserved the core formats and software for reporting acute childhood leukemia and therefore the extension to other cancer types had a shorter learning curve for the surveillance system´s personnel in municipalities. During the study period, notification of non-leukemia cancer were reported for 432 municipalities in almost all departments and districts (including municipalities with predominantly rural remote areas), which support the wide coverage of the surveillance system.

 For a previous study, we conducted a comparison between national high-cost account registry and SIVIGILA report during 2016 and found that 1394 incident cases of childhood cancer were identified and 1206 (86.5%) of them were reported to SIVIGILA, indicating that the systems captured 83% of all incident cases of childhood cancer in Colombia, which included non-leukemia cases. The 188 cases missing in SIVIGILA corresponded to different cancer diagnosis and municipalities distributed in 28 departments across the country. Therefore, we assumed that the presence of underreporting it is not concentrated in specific areas of the country and underreporting although is present, might not be the main explanation for the spatial heterogeneity in our results. However, health care access to cancer diagnosis is limited to specific regions in the country located in the main capital cities in Colombia and therefore delay in diagnosis (and derived delayed in reporting) might be present in remote semirural and rural municipalities. The analysis was conducted for 2014-2017 and databases were consolidated in 2019, therefore cases with delayed diagnosis had the opportunity to be included in the last two years as SIVIGILA required the reporting of incident and prevalent cases since 2014 for non-leukemia cases. However, the cases with missing diagnosis due to limitations in access to health care might be still present in the study but cannot be quantified.

 We have summarized and added these points or potential underreporting and underdiagnosis in the discussion section.

  

- A large cluster of childhood leukemia was detected in the center of Colombia. Can it be related to the large cluster of non-leukemia cases reported in the present study?

**Author's response to reviewer:**

 Thank you for your comment. One cluster for childhood leukemia was also identified in the center of the country. Clusters for both leukemia and non-leukemia cases might be related to each other, however the non-leukemia cluster is larger (327 municipalities compared with 109 identified in the leukemia cluster), more expanded to the North of the leukemia cluster, and with higher incidence rates located in municipalities with predominant rural areas.  We have added this comment in the discussion section.

  

- The four spatial clusters detected in the study were located in two distinct regions, as three clusters overlapped. The space-time clusters corresponded to the same spatial areas. In all, as a conclusion, I would say that only two clusters were detected (maybe over 2 years only) and I suggest to discuss the fact that the cluster frontiers were difficult to delineate (a wellknown limit of the cluster detection methods). The size of the largest detected cluster, in the central region, and the magnitude o the relative risk should also be discussed. A relative risk of about 2 in such a large area is quite surprising and unexpected. If an infectious or environmental factor was responsible for such an excess, it would have to be strongly associated to the risk of non-leukemia cancer and highly prevalent in the cluster area (in comparison to the remaining part of Colombia).

**Author's response to reviewer:**

 Thank you for your comment. We agree with your comment about the overlapping of clusters and concentration of cases in two clusters (rather than four) with the first expanded in a large are in the central region with a high relative risk. We have corrected this aspect in the abstract and conclusion and added a more detailed comment on the limitation to delimited clusters in the central region in the discussion section.

 ○ The statistical methods used in this study, Moran’s test for spatial autocorrelation and the Kulldorff’s scan method for cluster detection, were appropriate. However, some details on the methodology and the paramete,rs used could be added. In particular, the authors could add a reference for the Moran’s test for spatial autocorrelation, and explain how the neighborhood was defined in the study. Were two municipalities considered neighboring areas if the distance between their centroids was below a given threshold or if they shared a common border?

**Author's response to reviewer:**

 Thank you for your comment. The analysis considered neighboring based on the distance between the municipality´s centroids based on the Euclidean distance measured between two centroids of municipalities (with no threshold specification). We have added this comment in the methods section.

 ○ It would also be useful to provide further details on the scan method (estimation of a likelihood ratio for each window and selection of the most likely cluster, i.e. the window associated to the maximum ratio), and the simulations that were conducted to evaluate the significance thresholds (how many simulations were done?).

**Author's response to reviewer:**

 Thank you for your comment. The selection of the most likely cluster was selected based on the p-value of the log likelihood ratio (p>0,05 was considered statistically significant) and 999 replications were used in the simulation to evaluate the significance of the inference. We have added this comment in the methods section.

- Several other studies should be referred to in the introduction and discussion sections to provide more accurate information on childhood cancer etiology and the literature on childhood cancer clusters.

○ (Suggested publications):

 Etiology:

 'Genetic and nongenetic risk factors for childhood cancer' by Spector et al. (20151 ○ ). 

 'Environmental Exposure and Risk of Childhood Leukemia: An Overview' by Schüz et al. (20162). 

 ○ Clustering:

 'Childhood cancer trends in a western Canadian province: a population-based 22-year retrospective study' by Rosychuk et al. (20103).

 ○ 'Spatial clustering and space-time clusters of leukemia among children in Germany, 1987- 2007' by Schmiedel et al. (20074).

 ○ 'Leukemia and lymphoma incidence in children in Alberta, Canada: a population-based 22- year retrospective study by Kulkarni et al. (20115).

  ○ 'A cluster analysis of Pediatric Cancer Incidence Rates in Florida: 2000-2010' by Amin et al. (20146). 

 ○ 'Spatial and temporal variations of childhood cancers: Literature review and contribution of the French national registry' by Goujon et al. (20187).

**Author's response to reviewer:**

 Thank you for your suggestions. We have added the suggested references in the manuscript.

Minor comments:

 Abstract:

 "A sensitivity analysis was conducted with different upper limit parameters for the at-risk population." The upper limit was for the at-risk population included in the cluster.

**Author's response to reviewer:**

 Thank you for your correction. We have edited the sentence.

 ○ There are 1122 municipalities nationwide. Several municipalities had no observed cases, so that the cases were actually distributed in 432 different municipalities. However, all the municipalities were included in the analyses (if the at-risk population was not null). I suggest therefore to report that "2006 cases were distributed in 1122 municipalities".

**Author's response to reviewer:**

 Thank you for your comment. We have edited the sentence specifying the 1122 municipalities in the methods and the identification of cases in 432.

 ○ Regarding CNS tumors, it would be informative to specify whether non malignant cases were included.

**Author's response to reviewer:**

 Thank you for your comment. According to SIVIGILA protocol, CNS tumors include malignant and non-malignant cases. We have added this specification in the methods section.

 ○ Conclusion: differences in case registration should also be considered as a possible explanation.

**Author's response to reviewer:**

 Thank you for your suggestion. We have added that differences in case diagnosis in remote rural areas should also be considered as a possible explanation.

 Introduction:

 Based on reference 1, "The mean annual incidence of CC was estimated at 140.6 cases per million children". This incidence rate was estimated worldwide, which could be specified.

**Author's response to reviewer:**

 Thank you for your suggestion. We have edited the sentence.

 ○ "There are several conditions that have been identified as risk factors". The factors cited in this sentence were associated with childhood cancer with different degree of evidence. Some factors are considered as known (high dose ionising radiation, chemotherapy, certain genetic syndromes and some genetic polymorphisms, some viruses in lymphomas) or highly suspected (domestic and occupational parental exposure, socioeconomic conditions, infections and immune system stimulation for leukemia, birth weight, benzene exposure, air pollution) risk factors, while for other factors the literature is more heterogeneous and no firm conclusion can be drawn to date (tobacco and alcohol consumption). It is important to consider that point when discussing the etiology.

**Author's response to reviewer:**

 Thank you for your suggestion. We have added this comment in the introduction section for possible etiology for clusters.

 The following sentence is quite long and difficult to understand: "contributing to the generation of hypotheses about possible etiologies. Spatial analysis has been previously used for the study of CC, mainly for studying the geographical distribution of leukemias, since this type of analysis allows for the identification of space and time variations in a geographical area that generate clusters that indicate an increase in the tendency of the cases".

**Author's response to reviewer:**

 Thank you for your comment. We have edited the sentence.

 ○ At the end of the introduction, several studies on childhood cancer cluster detection are cited, but only one reference (ref 5) is provided for non-leukemia cancers. Maybe some of the references I reported above could be added here.

**Author's response to reviewer:**

 Thank you for your comment and suggested references. We have added some of the suggested references for non-leukemia cancers

 Methods:

 CC cases were identified by the National Surveillance System for Public Health, which registers "the newly confirmed and probable cases of CC". It is unclear to me what "probable cases" means? It seems that CC diagnoses are confirmed on the basis of diagnostic exams and coded according to the ICCC-3, so could the authors explain what are the probable cases that are registered (if not confirmed why are they registered?).

**Author's response to reviewer:**

 The SIVIGILA protocol for childhood cancer includes the case definition of “probable” when first clinical diagnosis is given and the confirmation of cases should be reported in a maximum of four weeks. All included cases in the analysis are confirmed cases. We have added a clarification in this sentence.

 ○ Are non-malignant CNS tumors registered in SIVIGILIA, and included in the study? Based on the number of CNS tumor cases reported in the result section (17.9% of non-leukemia cases), I assume that only malignant CNS tumors were included in the study. This information should be added in the main text and the abstract

**Author's response to reviewer:**

 Thank you for your comment. According to SIVIGILA protocol, CNS tumors include malignant and non-malignant cases. We have added this specification in this section.

 Statistical analysis:

 "We performed a descriptive analysis calculating frequencies and central tendency measurements." I don’t understand what "central tendency measurements" refers to?

**Author's response to reviewer:**

 Thank you for your comment. We refer to summary and dispersion measurements (i.e. mean and standard deviation), however in reported results we are providing only percentages so we agree on eliminating the word. We have edited the sentence specifying frequencies and percentages.

 ○ I understand that standardized rates were considered to account for potential differences in the age distribution of the pediatric population between municipalities. Were those potential differences also accounted for in the SaTScan analyses?

**Author's response to reviewer: **No, the cluster analyses were conducted with total population by municipality and year.

 Results:

 731 cases were excluded from the analyses, of which 57 had an unknown municipality of residence. It would be interesting to describe those cases in terms of type of diagnosis, year of diagnosis. Could the authors get other geographic information related to the area of residence? Were those cases grouped in a particular region?

**Author's response to reviewer:**

 Thank you for your comment. The cases were reported with codification for department with no specification of municipality. The 57 cases belonged to 20 departments distributed across the country (Atlántico, Magdalena, Meta, Cesar, La Guajira, Valle, Tolima, Antioquia, Cundinamarca, Huila, Caquetá, Casanare, Amazonas, Chocó, Putumayo, Cauca, Santander, Bolívar, Norte de Santander y Córdoba).

 We have added this information in the results section.

 ○ It would be useful to describe the distribution of the pediatric population in the 1122 municipalities to illustrate the potential heterogeneity.

**Author's response to reviewer:**

 Thank you for your comment. We have added a sentence describing the childhood population by municipaliy. (mean 9,880, median 3,336, minimum 149 in La Guadalupe municipality of Guainía and maximum 1,381,081 in Bogotá, the capital district)

  ○ The annual incidence rate for non leukemia cancer (44 cases/million) seems to be quite low compared to the overall incidence rate reported in Stealiarova-Foucher et al. 2017 for South America (133.9 cases/million, table S3), even if leukemia were excluded, and compared to the range reported in figure 2 legend ("more than 349 cases/million in the highest category"). This point needs clarification

 ○ The scan method selects the most likely cluster on the basis of a likelihood ratio that is calculated for each spatial window (from one municipality to the maximum size defined by the user). Likelihood ratios are very similar between two consecutive windows (as only one municipality or a small number of municipalities is added to the window at each step) that’s why overlapping significant clusters can be detected (as in this study). Wouldn’t it be interesting to run the scan method to detect non-overlapping cluster (this is an option in SaTScan)?

**Author's response to reviewer:**

 Thank you for your comment. We have run the analysis with no overlap and found two spatial clusters. Therefore, results are presented for both analysis concluding the presence of two clusters.

 Figure 2 is not as clear as figure 5. The four clusters can’t be identified precisely. Presenting one map for each cluster may be useful (or just 3 overlapping circles around the detected cluster areas in the central area and another circle centered on Cali).

**Author's response to reviewer:**

 Thank you for your comment. Figure 2 is showing rates by municipality and therefore the clusters are not identified and they are shown in figure 3 (previous figure 5).

 Discussion:

 The study cited in reference 18 focused on space-time clustering not on cluster detection. The authors reviewed the studies which tested for a space-time interaction, i.e. a general tendency of childhood cancer cases to occur more closely in space and time than expected under independent spatial and temporal patterns. This issue is really different from the question of detecting localized excesses of cases. The difference between space-time interaction and cluster detection (and even spatial clustering) should be clearly stated when presenting the results from reference 18.

**Author's response to reviewer:**

 Thank you for your comment. We have complemented the paragraph and made this clarification.

 ○ Regarding the heterogeneity of the previous study results on CC cluster detection, I agree with the authors that considering count data in geographical units or individual point data to detect localized clusters may lead to different conclusions, and may explained some differences between study results (end of page 7). However, I wouldn't say that the ecological approach is more sensitive than the point analysis on the basis of a small number of studies.  It may be that some excesses actually existed in some countries in some particular time periods (not necessarily related to an environmental factor), while cases were more homogeneously distributed in other countries. Sensitivity refers to situations of true excesses.

**Author's response to reviewer:**

 Thank you. We agree with your comment. We have edited the sentence.

 ○ At the end of the discussion, the authors noted "that in the SNCCC could exist some level of sub-registry caused by the limitation of the access to the health care services, especially in rural and isolated areas. "Again, this point is really important for the interpretation of the results (already noted as a major comment).

**Author's response to reviewer:**

 Thank you. We have added this aspect in abstract and conclusions and it is better explained in the discussion section.

 References

 1. Spector LG, Pankratz N, Marcotte EL: Genetic and nongenetic risk factors for childhood cancer. Pediatr Clin North Am. 2015; 62 (1): 11-25 PubMed Abstract | Publisher Full Text

 2. Schüz J, Erdmann F: Environmental Exposure and Risk of Childhood Leukemia: An Overview.Arch Med Res. 47 (8): 607-614 PubMed Abstract | Publisher Full Text

 3. Rosychuk RJ, Witol A, Stobart K: Childhood cancer trends in a western Canadian province: a population-based 22-year retrospective study.Pediatr Blood Cancer. 2010; 55 (7): 1348-55 PubMed Abstract | Publisher Full Text

 4. Schmiedel S, Blettner M, Kaatsch P, Schüz J: Spatial clustering and space-time clusters of leukemia among children in Germany, 1987-2007.Eur J Epidemiol. 2010; 25 (9): 627-33 PubMed Abstract | Publisher Full Text

 5. Kulkarni K, Stobart K, Witol A, Rosychuk RJ: Leukemia and lymphoma incidence in children in Alberta, Canada: a population-based 22-year retrospective study.Pediatr Hematol Oncol. 2011; 28 (8): 649-60 PubMed Abstract | Publisher Full Text

 6. Amin R, Hendryx M, Shull M, Bohnert A: A Cluster Analysis of Pediatric Cancer Incidence Rates in Florida: 2000–2010. Statistics and Public Policy. 2014; 1 (1): 69-77 Publisher Full Text 7. Goujon S, Kyrimi E, Faure L, Guissou S, et al.: Spatial and temporal variations of childhood cancers: Literature review and contribution of the French national registry.Cancer Med. 7 (10): 5299-5314 PubMed Abstract | Publisher Full Text.

# Peer review: Reviewer response for version 1

**DOI:** 10.5256/f1000research.30704.r81741

**Published:** 2021-04-15

**Authors:** Smita Asthana

**Affiliations:** 1 Division of Epidemiology and Biostatistics, Institute of Cytology and Preventive Oncology, NOIDA, New Delhi, Delhi, India

It’s a well written paper, a nationwide summarization of non-leukemia childhood cancer cases clustering in Colombia from 2014 to 2017. The topic is important. I recommend indexing with minor changes. My suggestions as given below.

1. Since it’s a study on non-leukemia cases this should be reflected in title of the manuscript also. Instead of 'childhood cancer' add 'non-leukemia childhood cancer'.
2. Introduction section second line, first paragraph “Childhood cancer (CC) …….. in this population.” Define the population instead of 'this population'.
3. Reference 2 can be given in English.
4. Introduction section, paragraph 1 line 5, for reference 2 please check the statement "The world health organization (WHO) estimates that nearly 300,000 new cases of CC are diagnosed every year in children between 0 and 19 years of age”. The article that the authors are referring to is giving the incidence as 400,000 and not 300,000, and age group is also given different in this WHO fact sheet.
5. Method section - Its better to give reference for International childhood cancer third edition.
6. It would be better if the authors discuss in a few lines the implications of the study.

Is the work clearly and accurately presented and does it cite the current literature?

Yes

If applicable, is the statistical analysis and its interpretation appropriate?

Yes

Are all the source data underlying the results available to ensure full reproducibility?

Yes

Is the study design appropriate and is the work technically sound?

Yes

Are the conclusions drawn adequately supported by the results?

Yes

Are sufficient details of methods and analysis provided to allow replication by others?

Yes

Reviewer Expertise:

NA

I confirm that I have read this submission and believe that I have an appropriate level of expertise to confirm that it is of an acceptable scientific standard, however I have significant reservations, as outlined above.

# Response

**Published:** 2021-05-24

**Authors:** Edgar F. Manrique-Hernández

**Affiliations:** Universidad Industrial de Santander, Colombia

**Clustering of childhood cancer in Colombia: a nationwide study**

**Reviewer #2: **Smita Asthana. Division of Epidemiology and Biostatistics, Institute of Cytology and Preventive Oncology, NOIDA, New Delhi, Delhi, India

  

1. Since it’s a study on non-leukemia cases this should be reflected in title of the manuscript lso. Instead of 'childhood cancer' add 'non-leukemia childhood cancer'.

**Author's response to reviewer: **Thank you for your detailed review and comments. We have edited the title

- Clustering of non-leukemia childhood cancer in Colombia: a nationwide study.

1. Introduction section second line, first paragraph “Childhood cancer (CC) …….. in this population.” Define the population instead of 'this population'.

**Author's response to reviewer: **Thank you for your detailed review and comments. This sentence uses the term "this population" since we previously referred to "children and adolescents" in the first line. We consider removing "in this population" from the sentence to avoid confusion for readers.

1. Reference 2 can be given in English

**Author's response to reviewer: **Thank you for your detailed review and comments. We have eliminated reference 2 (WHO fact sheet) as the source of data is the study reference 1.

1. Introduction section, paragraph 1 line 5, for reference 2 please check the statement "The world health organization (WHO) estimates that nearly 300,000 new cases of CC are diagnosed every year in children between 0 and 19 years of age”. The article that the authors are referring to is giving the incidence as 400,000 and not 300,000, and age group is also given different in this WHO fact sheet.

**Author's response to reviewer: **Thank you for your detailed review. We have corrected the number (400,000 new cases) and checked the age range is correct according to the WHO fact sheet and reference 1.

1. Method section - Its better to give reference for International childhood cancer third edition.

**Author's response to reviewer: **Thank you for your comment. We have added the suggested reference in the manuscript.

1. It would be better if the authors discuss in a few lines the implications of the study.

**Author's response to reviewer: **Thank you for your comment. We have added the implications of the study and limitations of data and methods in the discussion section.

# Peer review: Reviewer response for version 1

**DOI:** 10.5256/f1000research.30704.r79234

**Published:** 2021-03-04

**Authors:** Lisandro Agost

**Affiliations:** 1 Centro de Ecología y Recursos Naturales Renovables (CERNAR) – IIByT CONICET-UNC, Córdoba, Argentina

The article attempts to identify non-leukemia childhood cancer clusters (population under 15 years of age), across Colombia between 2014 and 2017. It uses data from official government sources, both disease and population, at the municipal level.

 Overall the article is clear and concise, perhaps too concise on some points. It is important research but I would consider the following changes and suggestions before indexing:

1. The title is omitting information as it is not a study of all types of cancers. It should clarify that it is about non-leukemia childhood cancer.

**Introduction section:**

1. *The mean annual incidence of CC was estimated at 140.6 cases per million children between the age 0–14 years in the period of 2001 to 2010. *A geographical reference is missing - worldwide or what?
2. *The world health organization (WHO) estimates that nearly 300,000 new cases of CC are diagnosed every year in children between 0 and 19 years of age. *Although the data is important, perhaps it would be better to obtain it for the 0-15 age group in order to be more coherent in the text.

**Methods section:**

1. *Colombia is a country located in the north of South America... *Description is too long, make a map of the study area or use the standardized rate map (Figure 2).
2. *Women make up 51.2% of the population, and children under the age of 15 years make up 22.6% compared to adults over the age of 65 years which represent 9.1%.***** It is not clear why the data is divided in this way. Why don't the authors put the total female - male population and then only the population under 15 (divided into female and male if want)?
3. *The global Moran index was calculated to estimate the spatial autocorrelation.* It is not clear whether they do this statistical analysis in STATA or with what software.
4. *We used an upper limit of the population at risk of 25% and for a sensitivity analysis we assess the results using upper limits of 50% and 10%. *I would explain in a little more detail what this analysis is for and why it is done.

**Results section:**

1. It would be interesting to show in this section (or in the annexes) a table with the distribution of cases per year, percentage of each group, crude and standardized rate, divided by ICCC 3 group (including leukaemias).
  Another interesting table, if it is not too big, would be to show the results of the distribution of cases per department, per year.
2. In general, none of the graphs or maps show the data sources. I don't know if this is a journal rule or if the authors omitted them.
3. Figures 3 and 4 are not useful. They should be redrawn showing more clearly the units of analysis (municipalities), and the clusters individually. For figure 3, for example, I suggest making four enlarged maps (from the general map of Colombia) showing the departments with the number of municipalities that include each cluster. Or simply show the municipalities included in the cluster. Why not use a map similar to the one in figure 2 or 5?

**Discussion section:**

1. In general, I feel that the discussion of the results is not enough. The authors should expand it, taking into account that they found numerous clusters, with important associated indicators.
  At no point is there an in-depth discussion of the results of this research in relation to the findings of previous research on leukaemia clusters (Space-time clustering of childhood leukemia in Colombia: A nationwide study, 2020).
  It would be very interesting to complement these studies and discuss what they have in common.
2. *The spatial studies based on the analysis of areas (ecological approach) such as this study, and the studies in Canada and in Palestine ^20– 22^,*...
  There seems to be an error in the text or in the citation.
3. It should be made explicit or discussed why the authors used only this cluster analysis method (Kulldorf’s) and not others. Also, what other statistical methods the authors believe could complement, in future research, the results obtained.
4. *Non-leukemia CC clusters identified in Colombia are located mainly in the central region of the country near the mountain ranges... *In this paragraph, I believe that the discussion of possible risk factors or associated risk factors for this disease should be expanded a little further and supported by literature from other research, reports or reviews. While the research does not set out to study causality, it can provide new lines of research by collecting and discussing possible risk factors.
  The bibliography of the risk factors mentioned above should be expanded.
5. I did not find the definition of SNCCC.
6. The discussion of limitations is poor. They should be expanded, better explaining the limitations and scope of the methodologies used and the results obtained.

**Conclusion section:**

1. Since the conclusion focuses on the possible factors associated with the results obtained, it seems important to me to develop these topics further in the Discussion section.

Is the work clearly and accurately presented and does it cite the current literature?

Yes

If applicable, is the statistical analysis and its interpretation appropriate?

Yes

Are all the source data underlying the results available to ensure full reproducibility?

Partly

Is the study design appropriate and is the work technically sound?

Yes

Are the conclusions drawn adequately supported by the results?

Partly

Are sufficient details of methods and analysis provided to allow replication by others?

Partly

Reviewer Expertise:

Ecology - Epidemiology - Childhood Cancer - Pesticide Risk Indicators

I confirm that I have read this submission and believe that I have an appropriate level of expertise to confirm that it is of an acceptable scientific standard, however I have significant reservations, as outlined above.

# Response

**Published:** 2021-05-24

**Authors:** Edgar F. Manrique-Hernández

**Affiliations:** Universidad Industrial de Santander, Colombia

**Clustering of childhood cancer in Colombia: a nationwide study**

**Reviewer #1**:  Lisandro Agost. Centro de Ecología y Recursos Naturales Renovables (CERNAR) – IIByT CONICET-UNC, Córdoba, Argentina.

 The article attempts to identify non-leukemia childhood cancer clusters (population under 15 years of age), across Colombia between 2014 and 2017. It uses data from official government sources, both disease and population, at the municipal level.

 Overall the article is clear and concise, perhaps too concise on some points. It is important research but I would consider the following changes and suggestions before indexing:

  

1. The title is omitting information as it is not a study of all types of cancers. It should clarify that it is about non-leukemia childhood cancer.

**Author's response to reviewer:** Thank you for your detailed review and comments. We have edited the title.

- Clustering of non-leukemia childhood cancer in Colombia: a nationwide study.

**Introduction section:**

1. The mean annual incidence of CC was estimated at 140.6 cases per million children between the age 0–14 years in the period of 2001 to 2010. A geographical reference is missing - worldwide or what?

**Author's response to reviewer:** Thank you for your detailed review and comments. We have added the clarification to worldwide reference in the manuscript.

1. The world health organization (WHO) estimates that nearly 300,000 new cases of CC are diagnosed every year in children between 0 and 19 years of age. Although the data is important, perhaps it would be better to obtain it for the 0-15 age group in order to be more coherent in the text.

**Author's response to reviewer:** Thank you for suggestion. The data is given by WHO for that range of age in the reference material. However, attending your comment on age range we modified the order of this sentence and it is now presented first and following information is related only to age range 0-14 years.

**Methods section:**

1. Colombia is a country located in the north of South America... Description is too long, make a map of the study area or use the standardized rate map (Figure 2).

**Author's response to reviewer: **Thank you for your suggestion. We have edited the population paragraph eliminating the details of geographical limits.

1. Women make up 51.2% of the population, and children under the age of 15 years make up 22.6% compared to adults over the age of 65 years which represent 9.1%. It is not clear why the data is divided in this way. Why don't the authors put the total female - male population and then only the population under 15 (divided into female and male if want)?

**Author's response to reviewer: **Thank you for your detailed review and comments. We have edited the sentence in the manuscript.

1. The global Moran index was calculated to estimate the spatial autocorrelation. It is not clear whether they do this statistical analysis in STATA or with what software.

**Author's response to reviewer:** Thank you for your detailed review and comments. We have added the clarification or ArcGIS use in the manuscript.

1. We used an upper limit of the population at risk of 25% and for a sensitivity analysis we assess the results using upper limits of 50% and 10%. I would explain in a little more detail what this analysis is for and why it is done.

**Author's response to reviewer:** Thank you for your suggestion. We have edited the sentence clarifying the it was conducted to identify consistency of clustering results across different upper limits.

 Results section:

1. It would be interesting to show in this section (or in the annexes) a table with the distribution of cases per year, percentage of each group, crude and standardized rate, divided by ICCC 3 group (including leukaemias). Another interesting table, if it is not too big, would be to show the results of the distribution of cases per department, per year.
2. In general, none of the graphs or maps show the data sources. I don't know if this is a journal rule or if the authors omitted them.

**Author's response to reviewer:** Thank you for your comments and suggestions. We have added Table 1 with the distribution of cases by sex, age group, year of diagnosis, and department or residence. Data source for childhood cancer is mentioned in the methods section (National Surveillance System for Public Health SIVIGILA, for its name in Spanish) but usually not included in maps as they are part of the manuscript.

  

1. Figures 3 and 4 are not useful. They should be redrawn showing more clearly the units of analysis (municipalities), and the clusters individually. For figure 3, for example, I suggest making four enlarged maps (from the general map of Colombia) showing the departments with the number of municipalities that include each cluster. Or simply show the municipalities included in the cluster. Why not use a map similar to the one in figure 2 or 5?

**Author's response to reviewer:** Thank you for your comment. We agree with your comment and have eliminated figures 3 and 4 as they show similar information in figure 5.

**Discussion section:**

1. In general, I feel that the discussion of the results is not enough. The authors should expand it, taking into account that they found numerous clusters, with important associated indicators. At no point is there an in-depth discussion of the results of this research in relation to the findings of previous research on leukaemia clusters (Space-time clustering of childhood leukemia in Colombia: A nationwide study, 2020). It would be very interesting to complement these studies and discuss what they have in common.

**Author's response to reviewer: **Thank you for your comment. We have expanded the discussion section pointing out the potential meaning of the results and their relation with findings of clusters of leukemia in Colombia.

1. The spatial studies based on the analysis of areas (ecological approach) such as this study, and the studies in Canada and in Palestine ^20–22^,... There seems to be an error in the text or in the citation.

**Author's response to reviewer: **Thank you for your detailed review. We have edited the sentence in the manuscript.

1. It should be made explicit or discussed why the authors used only this cluster analysis method (Kulldorf’s) and not others. Also, what other statistical methods the authors believe could complement, in future research, the results obtained.

 

**Author's response to reviewer: **Thank you for your comment. We have added a mention about the selection of method for cluster detection, its limitations and future analysis to complement data.

1. Non-leukemia CC clusters identified in Colombia are located mainly in the central region of the country near the mountain ranges... In this paragraph, I believe that the discussion of possible risk factors or associated risk factors for this disease should be expanded a little further and supported by literature from other research, reports or reviews. While the research does not set out to study causality, it can provide new lines of research by collecting and discussing possible risk factors. The bibliography of the risk factors mentioned above should be expanded.

**Author's response to reviewer: **Thank you for your comment. We have expanded the discussion of the potential factors that might be related to the clustering of non-leukemia childhood cancers in the center of the country, including infections, pesticides use, and other occupational and environmental risk factors for parents.

1. I did not find the definition of SNCCC.

**Author's response to reviewer:** Thank you for your detailed review and comments. We have corrected the acronym for SIVIGILA in the manuscript.

  

1. The discussion of limitations is poor. They should be expanded, better explaining the limitations and scope of the methodologies used and the results obtained.

**Author's response to reviewer: **Thank you for your comment. We have expanded the discussion of the limitations of the data sources and methodologies and the potential effects on the reported results.

 Conclusion section:

 Since the conclusion focuses on the possible factors associated with the results obtained, it seems important to me to develop these topics further in the Discussion section.

**Author's response to reviewer: **Thank you for your comment. The topics mentioned in the conclusion section are now better explained in the discussion section.
